# Arsenic: Various species with different effects on cytochrome P450 regulation in humans

**DOI:** 10.17179/excli2021-3890

**Published:** 2021-07-12

**Authors:** Mahmoud A. El-Ghiaty, Ayman O.S. El-Kadi

**Affiliations:** 1 Faculty of Pharmacy and Pharmaceutical Sciences, University of Alberta, Edmonton, Alberta, Canada

**Keywords:** arsenic, arsenic speciation, arsenic exposure, cytochrome P450, metabolism, xenobiotics

## Abstract

Arsenic is well-recognized as one of the most hazardous elements which is characterized by its omnipresence throughout the environment in various chemical forms. From the simple inorganic arsenite (iAs^III^) and arsenate (iAs^V^) molecules, a multitude of more complex organic species are biologically produced through a process of metabolic transformation with biomethylation being the core of this process. Because of their differential toxicity, speciation of arsenic-based compounds is necessary for assessing health risks posed by exposure to individual species or co-exposure to several species. In this regard, exposure assessment is another pivotal factor that includes identification of the potential sources as well as routes of exposure. Identification of arsenic impact on different physiological organ systems, through understanding its behavior in the human body that leads to homeostatic derangements, is the key for developing strategies to mitigate its toxicity. Metabolic machinery is one of the sophisticated body systems targeted by arsenic. The prominent role of cytochrome P450 enzymes (CYPs) in the metabolism of both endobiotics and xenobiotics necessitates paying a great deal of attention to the possible effects of arsenic compounds on this superfamily of enzymes. Here we highlight the toxicologically relevant arsenic species with a detailed description of the different environmental sources as well as the possible routes of human exposure to these species. We also summarize the reported findings of experimental investigations evaluating the influence of various arsenicals on different members of CYP superfamily using human-based models.

## 1. Introduction

Arsenic (As) is a naturally occurring element that is widely distributed in the environmental media. It is extremely toxic and does not seem to have any essential role in living organisms. The toxic nature of arsenic was recognized from early times, long before the documented recovery of its elemental form by the German alchemist; Albertus Magnus, amid the 13^th^ century (Meharg, 2005[248]).

Historically, arsenic was known as the “king of poisons” because of its wide use as a murder weapon. It was notorious for being specifically a “poison of kings” that was commonly used to assassinate rulers and nobility. This was attributed to the fact that arsenic compounds are usually tasteless and odorless and are also lethal at small amounts. Moreover, poisoning is also masked by non-specific symptoms that mimic those of food poisoning (Parascandola, 2012[288]). Being almost untraceable in the body, arsenic was frequently used as a poison till the 19^th^ century when a sensitive detection method was developed and published by the British chemist; James Marsh (Marsh, 1836[239]).

The first documentation of arsenic implication in cancer development dates back to early 1800s when John Paris noticed high rate of scrotal skin cancer among men working in copper smelting in Cornwall and Wales. These observations also included farm animals near the smelters. The British physician speculated that the exposure to arsenic fumes associated with the metals is the reason behind these findings (Bishop and Kipling, 1978[26]).

Because of its deleterious effects, arsenic is recognized as an environmental toxicant and carcinogen by regulatory agencies. Arsenic ranks first on the Substance Priority List (SPL) established by the Agency for Toxic Substances and Disease Registry (ATSDR). In this list, the substances posing significant potential threat to human health are prioritized based on their toxicity in addition to their frequency of occurrence and potential for human exposure (ATSDR, 2007[10]). Under the Canadian Environmental Protection Act (CEPA), arsenic and its compounds are included in the first Priority Substances List (PSL1) published in 1989 by Environment Canada and Health Canada. In 1993, environmental and human health assessment reports of the substances on this list revealed that arsenic and its inorganic compounds are toxic and pose a risk to the health of humans and to the environment (CEPA, 1993[46]). The Monographs Program of the International Agency for Research on Cancer (IARC), which identifies carcinogenic hazards to humans, has classified arsenic and its inorganic compounds as a Group 1 human carcinogen (IARC, 2004[164]).

With a varying degree of toxicity, arsenic has a wide range of trivalent and pentavalent compounds that fall under two main categories; inorganic compounds and organoarsenicals. From its natural repositories, arsenic is mobilized as water soluble inorganic species that can easily get into the food chain and undergo metabolic biotransformation yielding carbon-containing organic forms (Watanabe and Hirano, 2013[403]).

Arsenic toxicity is a complex and multifaceted process, and one important aspect of such toxicity is the interference with the metabolic machinery in the human body, with subsequent physiological derangements (Fu and Xi, 2020[110]). For instance, arsenicals, especially the trivalent species, are capable of disrupting the function of more than 200 enzymes (Rehman and Naranmandura, 2012[314]).

Cytochromes P450 (CYPs) represent a superfamily of hemoproteins that function as monooxygenases involved in the metabolic oxidation of a myriad of endogenous compounds as well as xenobiotics. Because of their considerable contribution in the metabolic system, their activity is regarded as a crucial element in the physiological homeostasis as well as the overall body exposure to foreign chemicals. Accordingly, alteration of such activity should have a direct impact on normal body function as well as the behavior of xenobiotics within the body (e.g. pharmacokinetics of an administered drug) (Nebert and Russell, 2002[276]).

In this regard, several studies have implicated arsenic and other heavy metals as modulators of CYPs regulation, which implies modification of their metabolic function (Anwar-Mohamed et al., 2009[6]). Characterizing the aspects of arsenic manipulation of CYP enzymatic system provides further insights into understanding the mechanisms underlying its toxicity which can be implemented in developing preventive strategies or exploited in treating certain illnesses.

This review offers a collective overview for the toxicologically relevant arsenic species and their origins. It also provides a detailed description of the different sources of arsenic release to the environment and discusses how humans can be exposed to such contaminant. Finally, it summarizes years of experimental investigations into the modulatory effects of various arsenic species on different members of CYP superfamily using human-based models.

A literature search was performed through MEDLINE database using the Medical Subject Headings (MeSH) term “Arsenic” combined with all of its subheadings. Additionally, a comprehensive literature review was conducted through Google Scholar and PubMed using search terms that included combinations of keywords such as; arsenic, history, chemistry, speciation, toxicity, poisoning, metabolism, methylation, arsenite, arsenate, arsenic trioxide, thioarsenicals, arsenobetaine, arsenocholine, arsenolipids, arsenosugars, arsines, trimethylarsine oxide, tetramethylarsonium ion, sources, environment, Canada, weathering, volcanoes, wildfires, mining, smelting, fuels, electronics, batteries, wood preservatives, pesticides, livestock, poultry, medication, treatment, cancer, exposure, drinking water, food, seafood, rice, cereals, air, pollution, cytochrome P450, CYP450, alteration, modulation, and regulation. Moreover, hand-searching was used to get additional relevant publications that are cited in previous review articles but were not retrieved through searching the electronic database. Full-text review was done after initial screening of all titles and abstracts as well as meticulous evaluation to include only articles published in peer-reviewed journals. Our search wasn't confined to a specific range of publication years.

## 2. Arsenic Chemistry and Speciation in Nature

### 2.1. Arsenic chemistry

Arsenic is found in the nature as a monoisotopic element (atomic number, 33; standard atomic weight, 74.92) and belongs to Group 15 of the Periodic Table. It is classified chemically as a metalloid because of having mixed properties of both metals and nonmetals; however, it is frequently referred to as a metal (Flora, 2015[104]).

Based on its electronic configuration, arsenic shows four common redox states: -3, 0, +3, and +5. Elemental arsenic (As^0^), also known as metallic arsenic, has three allotropes the most common of which is the steel-grey brittle solid polymorph. This pure form is rarely encountered in natural environment because of the inherent nature of arsenic to easily combine with other elements. The great ability of arsenic to lose electrons increases its cationic character, thus it can readily exhibit (+3) and (+5) oxidation states when combined with non-metals (most commonly oxygen and sulfur). The negative oxidation state (-3) arises when additional three electrons become more attracted towards arsenic upon interacting with less electronegative elements, basically metals, to form compounds known as arsenides (O'Day, 2006[283]).

Variable oxidation states of arsenic imply its affinity to participate in chemical bonding with other elements forming several compounds. There are over 300 naturally occurring arsenic minerals, which are mainly oxides and sulfides. Arsenic oxides and arsenosulfides may also contain other metals combined with arsenic. These minerals are considered valuable ore deposits if their copper, nickel, cobalt, or other metals can be economically recovered without negatively affecting the environment. Uncommon forms of natural arsenic minerals include metal arsenide and elemental arsenic (Drahota and Filippi, 2009[87]).

### 2.2. Arsenic species

#### 2.2.1. Arsenite and arsenate

Arsenic oxide minerals are either arsenite-containing minerals (arsenites) or arsenate-containing minerals (arsenates) and are formed naturally as secondary weathering products of other arsenic minerals. Arsenosulfides, arsenides, and elemental arsenic are commonly found associated with anoxic ore deposits, but once these minerals come in contact with oxygen, they are rapidly oxidized into arsenites (iAs^III^) and, in case of extensive oxidation, arsenates (iAs^V^) (Welch and Stollenwerk, 2003[408]). Oxidation is the initial step in the mobilization of arsenic from its deposits to the environment, and the rate of the process is much greater in the presence of water besides air (Jackson et al., 2003[170]).

**Figure 1:**
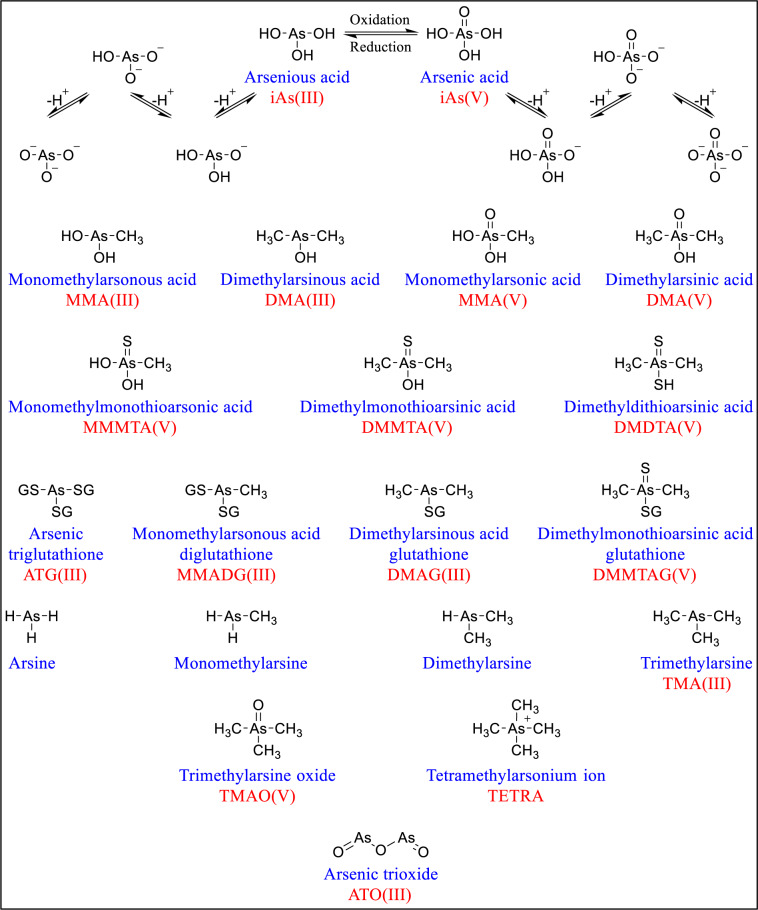
Chemical structures, names and abbreviations of some arsenic compounds. Arsenious and arsenic acids are interconverted under oxidizing and reducing conditions with subsequent dissociation of each acid to its respective oxo-anions by further increase in pH.

The hydrothermal fluids extract arsenic from its oxidized minerals as water-soluble species; trivalent arsenious acid (H_3_AsO_3_), pentavalent arsenic acid (H_3_AsO_4_) and their dissociated oxo-anions (Figure 1(Fig. 1)). Arsenic is transported in these fluids over long distances through extensive fractures in earth crust until they end up in ground water or reach the surface water. iAs^III^ and iAs^V^ are readily interconverted and the speciation of dissolved arsenic depends mainly on pH and redox potential, in addition to aqueous chemistry and biological activity (Shih, 2005[334]). In the reducing environments of hydrothermal fluids or anoxic groundwater, arsenic is predominantly in the form of arsenious acid, which exists as dissolved H_3_AsO_3_ at pH below 9.2 or as its dissociated oxo-anions (H_2_AsO_3_^-^, HAsO_3_^-2^, and AsO_3_^-3^) under more alkaline conditions. As arsenic-carrying fluids approach the earth surface and become diluted with aerated groundwater or reach surface water, iAs^III^ will begin to oxidize to iAs^V^. Eventually, arsenic acid becomes the dominant form under these oxidizing conditions, and then can be found as dissolved H_3_AsO_4_ at extremely acidic (pH <2) environment or as its associated anions (H_2_AsO_4_^-^, HAsO_4_^-2^) in less acidic or neutral conditions, or (AsO_4_^-3^) in alkaline waters (Mondal and Garg, 2017[264]; Smedley and Kinniburgh, 2002[344]).

#### 2.2.2. Methylated and thiolated arsenic species

Through water, these aqueous arsenic species can reach any life form. Once inside a living system, inorganic arsenic (iAs) can undergo extensive biotransformation, usually by methylation, into more complex organic compounds (oAs) (Challenger, 1945[48]; Hayakawa et al., 2005[139]; Naranmandura et al., 2006[272]). Figure 1(Fig. 1) lists the chemical structures, names and abbreviations of the major toxicologically relevant trivalent and pentavalent arsenic compounds. 

Each methylated species generated in the process could be excreted or remain in the organism and be further metabolized into more methyl-rich species. The most common methylated organoarsenicals include trivalent species as monomethylarsonous acid (MMA^III^) and dimethylarsinous acid (DMA^III^), in addition to pentavalent species such as monomethylarsonic acid (MMA^V^) and dimethylarsinic acid (DMA^V^) (Bentley and Chasteen, 2002[22]; Kumagai and Sumi, 2007[203]). 

**Figure 2:**
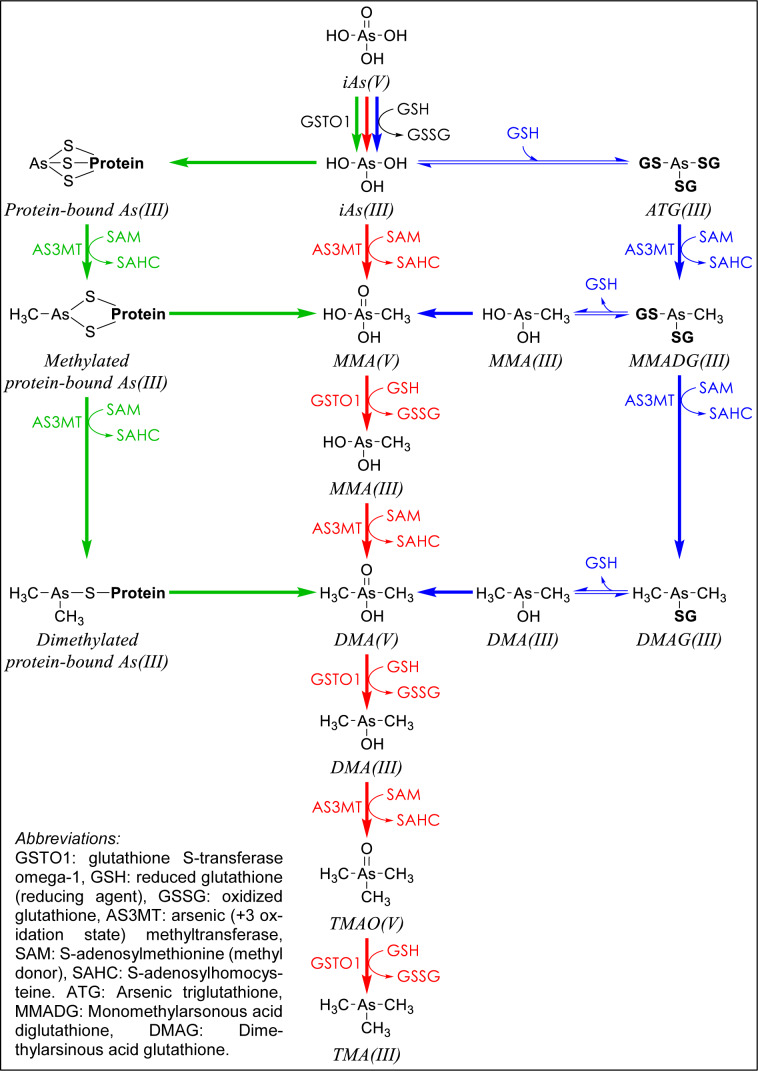
The three metabolic pathways proposed to explain arsenic metabolism via bio-methylation. Challenger's pathway (red arrows), Hayakawa's pathway (blue arrows), and Naranmandura's pathway (green arrows).

The exact reaction sequence and enzymes involved in arsenic biomethylation are still debated. Starting from iAs^V^, both tri- and penta-valent methylated arsenic species can be derived through three proposed mechanisms (Figure 2(Fig. 2)). The generally accepted classical pathway of Challenger (Challenger, 1945[48]) consists of two alternating steps of reduction as well as oxidation coupled with methylation. The reduction of iAs^V^ to iAs^III^ can be catalyzed by different enzymes with arsenate reductase activity such as glutathione S-transferase omega-1 (GSTO1), purine nucleoside phosphorylase (PNP), glyceraldehyde-3-phosphate dehydrogenase (GAPDH), and glycogen phosphorylase (GP), where glutathione (GSH) mediates the reaction for all of them except PNP for which the reductant is dihydrolipoic acid (DHLA) (Henke, 2009[146]). Hayakawa proposed an alternative pathway (Hayakawa et al., 2005[139]) mediated by non-enzymatic formation of trivalent arsenic-GSH complexes, which are sequentially methylated and subsequently hydrolyzed. Ultimately, the trivalent species are oxidized to the less toxic pentavalent counterparts. Based on the higher affinity of trivalent arsenicals to thiol group from proteins than that of GSH, a third pathway by Naranmandura (Naranmandura et al., 2006[272]) suggests that protein-bound trivalent arsenic is consecutively methylated in the presence of GSH. The end-products are the pentavalent species which are liberated from proteins upon oxidation of their corresponding trivalent forms.

In mammals, most of the absorbed inorganic arsenic in the body gets transformed and excreted in the urine as methylated metabolites (DMA > MMA), with a relatively little amount being excreted unchanged as iAs (Concha et al., 2002[66]). These urinary metabolites are mostly pentavalent species (DMA^V^ > MMA^V^) (Rehman and Naranmandura, 2012[314]). For instance, iAs-exposed human subjects have generally shown 10-30 % inorganic arsenic, 10-20 % MMA, and 60-80 % DMA in the urine (Vahter, 1999[384]). Interestingly, the pattern of iAs metabolism varies across mammalian species as a result of inter-species differences in the capacity to form various methylated arsenic metabolites, yielding eventually species-specific urinary profiles for these metabolites. Generally, the higher the methylation efficiency (towards forming DMA), the higher the excretion rate. In humans, the major component of urinary arsenic is DMA; however, the fraction of MMA in urine is relatively higher than that in other mammalian species; and that's probably why humans are more prone to arsenic toxicity than most experimental animals such as mice (Vahter, 1994[383]). The prominent efficiency of mice in arsenic methylation, as indicated by the high fraction of DMA and minimal amount of MMA in urine, results in a very fast urinary arsenic elimination (about 90 % of administered iAs dose is excreted within two days) (Vahter and Marafante, 1983[387]). On the other hand, lacking arsenic methylation ability in some species; such as marmoset and tamarin monkeys (Vahter and Marafante, 1985[388]; Vahter et al., 1982[389]; Zakharyan et al., 1996[438]), chimpanzees (Roy et al., 2020[317]; Vahter et al., 1995[385]; Wildfang et al., 2001[412]), and guinea pigs (Healy et al., 1997[142]); has been evidenced by the absence of methylated arsenicals in the urine after iAs treatment. This has been attributed to their inability to produce functional arsenic (+3 oxidation state) methyltransferase (AS3MT); the key enzyme in arsenic biomethylation. In these animals, iAs becomes strongly bound to different tissues in the body and gets excreted unchanged in the urine at a relatively much lower rate compared with other species, resulting in long retention time that leads to toxicity. Besides inter-species variability, intra-species variation in arsenic methylation and excretion patterns have been also reported in several arsenic-affected human populations. The reason behind such inter-individual differences may be one of several factors such as age, gender, length/intensity of iAs exposure, nutrition, or smoking (Vahter, 1999[382][384]). Additionally, both inter- (Drobná et al., 2010[88]) and intra- (González-Martínez et al., 2020[118]; Hernández et al., 2008[148]; Lu et al., 2018[226], 2019[227]; Meza et al., 2005[255]) species variations have been correlated to genetic factors affecting the expression of the enzymes involved in arsenic metabolic pathway especially AS3MT which, similar to other methyltransferases (Vahter, 2000[381]), has shown genetic polymorphisms.

The biomethylation process was widely regarded as a detoxification mechanism because, compared to iAs precursors, the pentavalent methylated forms; which are dominant, stable, and readily detected; are less reactive with tissue constituents and more easily excreted in urine resulting in lower retention of arsenic in the body. However, the discovery of trivalent methylated intermediates, which are less stable thus harder to be detected, has overthrown this assumption and rendered arsenic methylation a bioactivation process (Cullen, 2014[71]). Several studies have shown that MMA^III^ and DMA^III^ are more reactive and toxic than their pentavalent counterparts and even more than trivalent iAs (Khairul et al., 2015[187]; Petrick et al., 2000[296], 2001[297]).

In addition to methylated metabolites, several thiolated forms have been detected in mammals including humans. Thioarsenicals are sulfur-containing derivatives of the methylated oxo-arsenicals where the oxygen bonded to arsenic atom is replaced by sulfur, thus forming As-SH and/or As=S interchangeable tautomeric substructures (Herath et al., 2018[147]; Suzuki et al., 2010[358]). Only pentavalent, but not trivalent, thiolated metabolites have been identified in biological systems, such as monomethylmonothioarsonic acid (MMMTA^V^), dimethylmonothioarsinic acid (DMMTA^V^), and dimethyldithioarsinic acid (DMDTA^V^) (Sun et al., 2016[356]). Thioarsenials are suggested to be produced through enterohepatic circulation. Methylated species excreted in the bile get converted by gastrointestinal microbiota into thiolated forms, which are then absorbed into the blood and end up excreted in the urine (Bu et al., 2011[36]). 

When trivalent species, both inorganic or methylated, are introduced to the biological systems, trivalent thioarsenicals are hypothesized to only act as transient intermediates which eventually get oxidized to their pentavalent counterparts (Fan et al., 2018[100]). Because of their high affinity for sulfhydryl groups, trivalent arsenicals are usually bound *in vivo* to glutathione or proteins forming sulfur-containing complexes, which are not considered thioarsenicals. Examples of glutathione-conjugated arsenic species include arsenic triglutathione (As^III^-GS_3_), monomethylarsonous acid diglutathione (MMA^III^-GS_2_), and dimethylarsinous acid glutathione (DMA^III^-GS) conjugates (Ponomarenko et al., 2014[303]; Shen et al., 2013[333]). Interestingly, the thiolated arsenic metabolite, DMMTA^V^, was the only pentavalent species to be detected in a complex with glutathione. The formation of dimethylmonothioarsinic acid glutathione conjugate (DMMTA^V^-GS) is attributed to DMMTA^V^ affinity to interact with sulfhydryl groups of biomolecules such as glutathione, resulting eventually in profound oxidative stress rendering it the most cytotoxic among other thiolated metabolites and all pentavalent forms (Herath et al., 2018[147]; Naranmandura et al., 2011[270]).

In most natural waters that have detectable arsenic, inorganic As^III^ or As^V^ are dominant, while organoarsenicals are often absent or found in very low concentrations. Since the methylation of arsenic is exclusively biotic, the presence of oAs in water is associated with microorganisms such as bacteria and phytoplankton which can be mostly found in surface waters (Hasegawa et al., 2019[138]). The increased ratio of organic species in surface waters during summer may be explained by the enhanced methylation reactions catalyzed by microbial activity (Hasegawa et al., 1999[137]). On the other hand, the near absence of methylated forms in groundwater can be attributed to low populations of microorganisms there. However, oAs may be detected in groundwater if it was infiltrated with surface waters that already have such organic species (Mandal and Suzuki, 2002[235]). It is worth mentioning that abnormally high levels of oAs can be observed in areas that are impacted by industrial pollution and human-generated wastes.

#### 2.2.3. Arsenobetaine, arsenocholine, arsenolipids, and arsenosugars

In addition to accumulation of a minor percentage of methylated metabolites (Taylor et al., 2017[364]), iAs is mainly retained in marine organisms as more complex species of organoarsenicals. The most predominant of such species is arsenobetaine (AsB) which is found in the majority of finfish and shellfish. Chemically, arsenobetaine is an arsenic analog of the osmolyte glycine betaine (trimethylglycine), and such structural similarity suggests that it may have an osmotic role within marine animals (Popowich et al., 2016[304]). Arsenocholine (AsC) serves as a precursor which is readily converted to AsB (Francesconi et al., 1989[107]), thus it can be only detected at low levels in seafood (Kirby and Maher, 2002[192]; Suner et al., 2002[357]). However, AsC has been reported as a major arsenical in some sea anemones (Ninh et al., 2008[279]) and species of jelly fish (Hanaoka et al., 2001[130]). Arsenolipids (AsLipids) is another group of arsenic compounds that have been detected at low levels in marine life with significant fractions being generally associated with oily fish. The classes of these compounds include fatty acids (AsFAs), hydrocarbons (AsHCs), and phospholipids (AsPLs) (Taleshi et al., 2014[361]). Arsenosugars (AsSugars) are ribose derivatives representing arsenic species which are commonly found at major fractions in marine algae such as seaweeds (Xue et al., 2017[425]).

#### 2.2.4. Arsines

Arsines are a special family of volatile trivalent arsenic-bearing chemicals that comprises the inorganic arsine (AsH_3_) and the organic methylarsines; mono-, di-, and tri-methylarsine ((CH_3_)AsH_2_, (CH_3_)_2_AsH, and (CH_3_)_3_As) (Mestrot et al., 2011[251]). The discovery of gaseous arsenic dates back to 19^th^ century, when vivid green color of some arsenic compounds such as Scheele's green (copper arsenite) and Schweinfurt's green (copper acetoarsenite) was widely used as a pigment for dyeing fabrics and wallpaper. At that time, reported cases of child deaths and people suffering from chronic illness were linked to living in rooms decorated with As-pigmented wallpaper, especially when it gets damp in closed and poorly ventilated spaces (Bartrip, 1994[14]; Chasteen et al., 2002[54]).

The reason behind this was mistakenly believed to be the inhalation or ingestion of As-bearing particles released mechanically from the wallpaper. However, in late 1800s, Bartolomeo Gosio found that a toxic arsenic gas with a strong garlic-like odor was produced from the inorganic arsenic pigment by a fungus, *Penicillium brevicaule* (reclassified as *Scopulariopsis brevicaulis*), growing on damp wallpaper and feeding on starch adhesive (Gosio, 1892[122]; Thom and Raper, 1932[367]). The exact nature of this gas, eponymously named "Gosio gas", remained unclear until 1930s when Frederick Challenger et al. demonstrated that the gas was trimethylarsine (TMA) which is formed as an end product of arsenic methylation (Figure 2(Fig. 2)) (Challenger, 1945[48]; Challenger and Higginbottom, 1935[49]; Challenger et al., 1933[50]). Ever since, Challenger became a leader in the study of biomethylation and organometals. Mechanistically, how arsines are generated from non-volatile arsenic species remains unexplained. It is postulated that arsine is formed by the reduction of arsenite or arsenate, while for other arsines, the process involves formation of trivalent methylated arsenicals which, in case of (CH_3_)AsH_2_ and (CH_3_)_2_AsH, undergo additional hydride transfer yielding their volatile counterparts. Therefore, arsines are considered intermediates in arsenic biomethylation pathway with the end product being TMA (Mestrot et al., 2013[252]; Planer-Friedrich et al., 2006[302]).

Upon formation, these volatile compounds are partitioned from aqueous solutions into the atmosphere under ambient standard temperature and pressure conditions; therefore, they should not be confused with non-volatile arsenic species emitted from arsenic-bearing minerals to the atmosphere at high temperatures such as in volcanoes or smelters. The latter determines evaporated arsenic species that are condensed and adsorbed onto the particulate matter (Sanchez-Rodas et al., 2007[324]; Tirez et al., 2015[370]).

In nature, generation of arsines is mainly dependent on a biotic component. In such organisms, biovolatilization into gaseous species is considered as a mechanism of arsenic release thus alleviating its poisoning (Qin et al., 2006[308]; Yuan et al., 2008[435]). Arsines are produced under anaerobic conditions by microorganisms such as bacteria, fungi, methanoarchaea, protozoans, and algae (Wang et al., 2014[398]). Several studies have shown that arsenic volatilization can happen in humans via intestinal microbiota (Diaz-Bone and van de Wiele, 2009[82]; Michalke et al., 2008[256]; Van de Wiele et al., 2010[392]). Formation of volatile species through pre-systemic metabolism, mediated by gut microbial ecosystem, can modify arsenic toxicokinetics and total body exposure, with the impact on human health being determined by the relative toxicity of generated species compared to the ingested forms. The sources of volatile arsenicals include arsenic-bearing waste in landfills (Pinel-Raffaitin et al., 2007[301]), sewage sludge (Michalke et al., 2000[257]), soils and rice paddies (Mestrot et al., 2009[253], 2011[250]), and biogas digesters (Mestrot et al., 2013[254]). Additionally, TMA has been reported as the main volatile arsenic species in natural gas (Krupp et al., 2007[200]; Uroic et al., 2009[380]) and geothermal spring water (Planer-Friedrich et al., 2006[302]).

The relatively low levels of volatile species, compared to total arsenic, in natural environmental systems can be attributed not only to the limited arsenic biovolatilisation, but also to their poor atmospheric stability. Because of their reactive nature towards oxygen, arsine is directly oxidized to arsenite or arsenate, while mono-, di-, and tri-methylarsine are readily oxidized to the corresponding pentavalent methylated arsenic oxides; monomethylarsonic acid (MMA^V^), dimethylarsinic acid (DMA^V^) and trimethylarsine oxide (TMAO), respectively. These non-volatile oxidation products will be adsorbed onto atmospheric particles or, ultimately, find their way into rainwater (Haas and Feldmann, 2000[125]; Jakob et al., 2010[171]). Interestingly, some studies have demonstrated that these volatile species are quite stable in the environment (Mestrot et al., 2011[251]). Studying the environmental stability of volatile arsenicals is highly important, as it is a major determinant of their impact on the population. Higher stability of these compounds implies their travel over considerable distances, without chemical change, thus imposing threats not only in the area of emissions but also in remote locations. In such case, monitoring of global arsines' fluxes would be necessary.

#### 2.2.5. Trimethylarsine oxide

Aside from being the oxidation product of TMA, TMAO can be also produced either through microbial arsenic biomethylation (Cullen et al., 1979[74], 1994[72], 1995[73]), then it possibly undergoes subsequent reduction to volatile TMA (Pickett et al., 1981[300]), or as a degradation product of the main marine arsenical, AsB (Hanaoka et al., 1988[133], 1989[132], 1992[129][131], 1995[130]; Kaise et al., 1987[181]). AsB degradation accounts for its complete absence (Jenkins et al., 2003[175]) or very low levels (Glabonjat et al., 2018[116]) in seawater, despite being the predominant form in marine life which eventually gets released to water in considerable amounts from dead marine animals. Such microbial degradation is regarded as a part of arsenic cycling in marine ecosystem, and results in generating simpler species including TMAO (Suner et al., 2002[357]).

The formation of TMAO as a minor constituent in several marine animals (Norin et al., 1985[282]; Taylor et al., 2017[364]) can be mainly attributed to the presence AsB-degrading microorganisms inside these animals (Gailer et al., 1995[112]; Kaise et al., 1998[182]; Kirby and Maher, 2002[192]), but in some cases, it may result from biomethylation of inorganic arsenic by microbial activity in the gut of fish (Edmonds and Francesconi, 1987[93]; Maher et al., 1999[233]). TMAO formation has been reported in some terrestrial organisms as well (Braeuer et al., 2018[34]; Kuehnelt et al., 2000[201]).

In mammals, including humans, DMA^V^ is the end product of arsenic biomethylation (Rehman and Naranmandura, 2012[314]), with no (Hughes et al., 2000[161]; Naranmandura et al., 2010[271]) to minimal (Cohen et al., 2002[64]; Lu et al., 2003[228]; Yoshida et al., 1997[430], 1998[431], 2001[432]) detection of TMAO because of DMA^V^ rapid clearance that doesn't allow its further methylation to the trimethylarsinic form (Marafante et al., 1987[238]). Instead of mammalian hepatic metabolism, TMAO formation in mammals is believed to be achieved through a different arsenic methylating pathway mediated by intestinal microbial activity (Kuroda et al., 2001[207]). Gut metabolism of arsenic can go beyond TMAO to further transform it into volatile TMA (Pickett et al., 1988[299]) resulting in complete (Marafante et al., 1987[238]) or partial disappearance (Yoshida et al., 2001[432]) of TMAO in fecal samples. Such extrahepatic metabolism may explain why TMAO could not be found in liver tissue despite being detected in urine (Liu et al., 2015[223]). Additionally, TMAO formation is associated with oral arsenic administration (Cohen et al., 2002[64]; Lu et al., 2003[228]; Yoshida et al., 1997[430], 1998[431], 2001[432]), while complete absence of TMAO was observed in experiments studying arsenic through intravenous exposure (Hughes et al., 2000[161]; Naranmandura et al., 2010[271]). In a study by Yoshida et al., TMAO detection in urine after intraperitoneal injection of DMA^V^ can be attributed to background arsenic derived from fish which is a source of protein commonly found in the standard rodent dietary chow which was used in that study (Yoshida et al., 2001[432]). Comparing arsenic levels in rats feeding on standard rodent chow, in which AsB is the main chemical form of arsenic, with those feeding on arsenic-depleted rodent chow, in which casein is used instead of fish as the protein source, has demonstrated that diet can significantly contribute to arsenic exposure. In this case, AsB is the main form of arsenic excreted in urine, with trace amounts being in the form of TMAO (Kobayashi and Hirano, 2016[196]). TMAO can be also produced from breaking down ingested AsB by gut microbiome in humans (Harrington et al., 2008[136]) and other mammals (Yoshida et al., 1998[431], 2001[433]). Moreover, traces of TMAO have been detected in humans as an intestinal degradation product of AsSugars (Francesconi et al., 2002[108]). Additionally, tetramethylarsonium salt may also undergo microbial degradation yielding TMAO (Hanaoka et al., 1994[130]).

#### 2.2.6. Tetramethylarsonium ion

Tetramethylarsonium ion (TETRA) is a trace arsenic species that has been detected in aquatic (Lai et al., 1999[211]; Larsen et al., 1993[215]; Sloth et al., 2003[343]) as well as in some terrestrial organisms (Kuehnelt and Goessler, 2003[201]). Exceptionally higher percentages of TETRA are found in some marine species such as clams (Shiomi et al., 1987[338]), gastropods (Francesconi et al., 1988[106]; Ruiz-Chancho et al., 2013[318]), and annelids (Geiszinger et al., 2002[114]). It is postulated that TETRA production is mediated by microbial degradation of AsB (Suner et al., 2002[357]) and/or methylation of other arsenic species (Kirby and Maher, 2002[192]; Yoshida et al., 1998[431]).

### 2.3. Importance of arsenic speciation

Arsenic speciation is of great importance because the toxicity of this element is defined by species-related factors such as its oxidation state and molecular nature, that is, different forms of arsenic have vastly different toxicity on humans. Therefore, for a risk assessment, the identification of individual species would be more useful than the determination of total arsenic, which may overestimate harmful arsenic exposure. For example, inorganic species as arsenite or arsenate are well-recognized toxic and carcinogenic agents, while organic seafood-derived forms are fairly safe with no health risks posed on seafood consumers (ATSDR, 2007[10]). Since seafood accounts for the largest contribution to arsenic exposure, primarily in organic forms which are mainly excreted unchanged, misleading estimates of inorganic arsenic exposure may be drawn from measuring total arsenic after seafood consumption (Navas-Acien et al., 2011[274]). High seafood consumption has been associated with elevated total arsenic in urine, blood, and other parts of the body (Birgisdottir et al., 2013[25]; Miklavcic et al., 2013[259]). That is why participants in studies assessing arsenic exposure and its related health impact are instructed to refrain from eating seafood (Brima et al., 2013[35]), and in animal experiments, non-standard arsenic-depleted chow is used (Kobayashi and Hirano, 2016[196]). These food restrictions are usually applied before commencing the study to reduce any background levels of arsenic in the body.

## 3. Arsenic Sources in the Environment (with Examples from the Canadian Environment)

**Figure 3:**
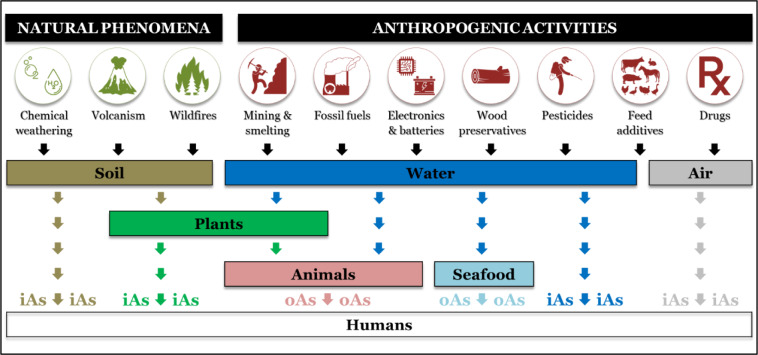
Pictorial depiction of various natural phenomena and anthropogenic activities that contribute to arsenic release from its natural repositories to the environment. Subsequently, human exposure to the released arsenic can take place either directly through soil, water, or air; or indirectly through different food products. The main arsenic form, inorganic (iAs) or organic (oAs), is shown for each route of exposure.

Arsenic is a natural component of the earth's crust, with varying amounts depending on local geological history of the geographic region. From its natural repositories, arsenic is released and dispersed into the pedosphere, hydrosphere, and atmosphere (Figure 3(Fig. 3)). Natural geogenic processes including weathering and volcanism achieve this release slowly. However, greatly enhanced release results from anthropogenic activities that involve arsenic-containing products or wastes. For instance, the global anthropogenic contribution to atmospheric emissions of arsenic is estimated to be about three times higher than that from natural sources (WHO, 2001[411]). It is of grave importance to understand how arsenic is introduced to the biosphere in order to characterize its environmental levels and subsequently assess the risk of human exposure.

### 3.1. Natural arsenic sources

#### 3.1.1. Chemical weathering

Chemical weathering in the presence of oxygen and water is the main natural mechanism of arsenic mobilization from its minerals. Arsenic-bearing minerals such as arsenopyrite (FeAsS), realgar (As_4_S_4_), and orpiment (As_2_S_3_) represent the starting point for the processes of oxidation and hydrolysis, from which arsenic is subsequently released resulting in enrichment of the surrounding soil with highly soluble species (Masuda, 2018[242]). The global average natural arsenic level released into uncontaminated soil is 5 mg/kg, with much higher levels being detected near high geological deposits of arsenic-rich minerals, or in human-impacted spots as mining areas (ATSDR, 2007[10]).

In Canadian uncontaminated soil, arsenic can be found naturally at levels of 4.8-13.6 mg/kg (Wang and Mulligan, 2006[399]). Pyrite oxidation, upon exposure to the air, in acid sulfate soils located in northwestern Alberta results in arsenic enrichment up to 37.9 mg/kg (Bennett and Dudas, 2011[21]). In British Columbia, Warren et al. have detected extremely high arsenic concentrations of 4600 mg/kg in A_2_ soil horizon in the neighborhood of some mineralized veins (Warren et al., 1964[401]).

#### 3.1.2. Volcanism

Volcanism is another significant natural arsenic-releasing mechanism. Large amounts of arsenic are mobilized, especially to the atmosphere, by volcanic activity through volcanic emissions including ash and gases (Matschullat, 2000[243]; Ng, 2005[277]; Signorelli, 1997[341]). In addition to ground water contamination by volcanic eruptions, surface water can be also affected by deposition and dissolution of volcanic ash (Juncos et al., 2015[180]; Morales-Simfors et al., 2019[265]).

#### 3.1.3. Wildfires

Wildfires represent an increasingly important global phenomenon, particularly tied to hot and dry weather, and their risk is expected to increase as a result of climate change (Finlay et al., 2012[103]). They contribute to releasing large quantities of toxic pollutants including arsenic (Johnston et al., 2019[177]; Makkonen et al., 2009[234]). Significantly higher levels of arsenic are detected in wildfire-impacted areas, especially in urban residential areas, because of burning buildings and other urban elements, compared to open wildlands (Wittig et al., 2008[414]; Wolf et al., 2011[415]).

In Canada, wildfire has been a major environmental concern for a long time, burning approximately 2 million hectares of forest annually (in some years, more than 7 million hectares) (Stocks et al., 2003[352]). For instance, in 2003, British Columbia had catastrophic wildfires where nearly 2,500 fires burnt more than 265,000 hectares (Beck and Simpson, 2007[16]). The costliest natural disaster in the history of Canada was the 2016 Horse River wildfire in Alberta. Because of the toxic fire ashes containing arsenic, the re-entry of Fort McMurray residents, who were evacuated from the wildfire-ravaged area, was delayed for five months. Fourteen months later, samples of ground ashes from wildland-urban interface fires in Fort McMurray have shown residual arsenic pollution originating, most probably, from burning local buildings rather than forests (Kohl et al., 2019[197]).

### 3.2. Anthropogenic arsenic sources

In addition to these natural processes, a wide range of human activities has been also implicated in arsenic mobilization. These activities, because of environmental awareness, have become historical and do not exist anymore, or, because of technological improvement and remediation, still exist but are well-regulated under rigorous restrictions for arsenic release. However, the old practices have resulted in the release of massive amounts of arsenic that have impacted the environment till today, because once released, arsenic cannot be destroyed but can only be converted into different forms thus spreading its toxic effects throughout the ecosystem (Leist et al., 2000[219]).

#### 3.2.1. Mining and smelting

The significant natural occurrence of arsenic in sulfide-bearing ore deposits of metals such as lead, copper, zinc, gold and silver, poses high risk of arsenic liberation upon extraction of such metals (Basha et al., 2008[15]). Mining and metallurgical processing operations (including comminution, disposal of mine wastes and tailings, smelting, and refining) represent a significant source of heavy metals pollution including arsenic (Razo et al., 2004[313]). Mining can accelerate the weathering process via oxidation of arsenic-bearing minerals, mainly sulfides, resulting in the formation of sulfuric acid. The outflow of such acidic water, namely acid mine drainage, with its elevated levels of heavy metals facilitates arsenic release to the soil in the vicinity of mines (Straskraba and Moran, 1990[354]). High concentrations of arsenic have been detected in the blood (Kesici et al., 2016[186]), urine (Dartey et al., 2013[75]), and hair (Murao et al., 2004[267]) of miners.

In smelters, the pyrometallurgical treatment of metal ores, such as copper, results in removal of arsenic, a common impurity in copper ores, by oxidation into arsenic trioxide (As_2_O_3_). Subsequently, under high temperatures, As_2_O_3_ volatilizes and escapes in the generated flue gases, and ultimately, as the gases cool down, condenses on particulate matter and is captured by the flue dust as white powder. Such dust not only affects the atmosphere, but also can deposit to contaminate soil and water (Weisenberg et al., 1979[407]). As_2_O_3_ can be found naturally as two dimorphs of trivalent arsenic oxide minerals (arsenites), namely; arsenolite and claudetite, but its common source is oxidation through roasting of arsenic-bearing ore minerals or coal. The gaseous emissions from copper smelters account for about half of the annual anthropogenic arsenic emissions to the atmosphere (Chen et al., 2012[57]). Besides atmospheric releases, wastewater from these smelters also contains considerable amounts of arsenic and must be treated before disposal (Hansen and Ottosen, 2010[134]). Exposure of smelter workers to arsenic results in high urinary concentrations of its metabolites (Vahter et al., 1986[386]) and it has been tied to peripheral neuropathy (Feldman et al., 1979[101]; Lagerkvist and Zetterlund, 1994[210]), Raynaud's phenomenon (Lagerkvist et al., 1986[209]), cancer (Enterline et al., 1995[97]), and other disorders (Axelson et al., 1978[11]). Arsenic-mediated lung cancer is identified as the major cause of mortality among smelter workers (Järup et al., 1989[174]; Lubin et al., 2008[229]), as suggested by high arsenic concentrations detected in autopsy samples of lung tissue from dead workers (Wester et al., 1981[409]). The carcinogenic effect of smelter-generated arsenic also extends to impact those who are living in the vicinity of smelters (Pershagen, 1985[292]).

Few kilometers away from Yellowknife (Northwest Territories), Giant Mine was a gold mine that operated for over five decades, until it became officially abandoned in 2005. Arsenopyrite-bearing gold ore mining operations, especially roasting, have swamped the surrounding environment with massive amounts of As_2_O_3_ dust from stack emissions. Moreover, thousands of tons of As_2_O_3_ were stored in underground chambers and are currently an ongoing source of arsenic to groundwater. A costly remediation plan to permanently freeze these chambers, to keep groundwater seepage out, was approved by the Canadian federal government in 2014 (Jamieson, 2014[172]).

Another example of legacy arsenic contamination is located in Cobalt town (Ontario) where historical silver and cobalt mining activity took place. The mineralogical association of arsenic with silver and cobalt ores resulted in tons of arsenic-rich tailings and wastes that were disposed into nearby depressions (often, lakes). Almost a century after ending the operations there, wastes are still lingering in both aquatic and terrestrial environments till today (Sprague and Vermaire, 2018[348]). In northern Saskatchewan, high levels of arsenic have been detected in Rabbit Lake uranium mine tailings (Moldovan et al., 2003[261]). Historical gold mining in Nova Scotia has left many arsenic-rich tailings deposits in different areas across the province (Walker et al., 2009[395]).

Athabasca oil sands, located in northeastern Alberta, are large deposits of bitumen that are considered the largest known reservoir of crude bitumen in the world. Being the largest in the world, surface mining operations in these bituminous sands result in generating massive volumes of wastes in which arsenic is present in significant levels, thus posing ecological risks. The development of mining operations in that area has been accompanied with increased arsenic concentrations in Athabasca River (Donner et al., 2017[86]).

Smelters across Canada pose a great threat to the environment through arsenic release. Examples include base-metal smelting complex in Flin Flon (Manitoba) and Creighton (Saskatchewan) (Zhang et al., 2009[442]), lead smelter in Belledune (New Brunswick) (Parsons and Cranston, 2006[290]), copper smelter in Rouyn-Noranda (Québec) (Bonham-Carter et al., 2006[29]), and lead-zinc processing facility, formerly a gold smelter, in Trail (British Columbia) (Caplette and Schindler, 2018[43]).

#### 3.2.2. Fossil fuels

As a fossil fuel, coal is combusted to produce very high temperatures used in several applications, most notably of which is generating electricity, through steam, in coal power stations. Coal is a natural source of arsenic and primarily responsible for its release in different forms. During combustion, only minor part remains in bottom ash, while the rest volatilizes and either escapes in gaseous phase or, mainly, deposits on fly ash (Yudovich and Ketris, 2005[436]). Because it occurs as a surface precipitate, arsenic in fly ash is highly leachable, thus ending up in soil or water (Mattigod et al., 1990[244]). Through technologies as electrostatic precipitators, more than 95 % of fly ash is collected before being released from smoke stacks, thus decreasing atmospheric emissions, however its subsequent disposal remains a threat to soil and water (Wang et al., 2018[396]). Metabolites of arsenic were detected in the urine of power plants' workers (Yager et al., 1997[426]). Moreover, arsenic release associated with coal combustion is strongly correlated to the incidence of cancer among these workers (Bencko et al., 2009[19]; Pesch et al., 2002[295]). Combustion of fuels in automotive engines can also contribute to arsenic emissions (Pulles et al., 2012[306]; Talebi and Abedi, 2005[360]).

Establishment of coal-fired power plants has resulted in enrichment of arsenic in Wabamun Lake (Alberta) sediments to concentrations beyond the lowest effects levels (LELs) for toxicity to benthic organisms (Donahue et al., 2006[85]). Compared to background areas, statistically significant higher concentrations of arsenic have been detected in Grand Lake (New Brunswick) sediments because of coal-combustion ash discharges (Lalonde et al., 2011[212]).

#### 3.2.3. Electronics and batteries

Arsenic is an important element in various industrial applications. It is a common n-type dopant in manufacturing semiconductors, with gallium arsenide (GaAs) being the second, after doped silicon, most commonly used semiconductor material in electronics industry such as integrated circuits (ICs), light emitting diodes (LEDs), laser diodes (LDs), and solar cells (Neamen, 2012[275]). GaAs and other arsenic-based III-V semiconductors, such as indium arsenide (InAs), may impose serious toxic and carcinogenic pulmonary effects on workers in the semiconductor industry (Tanaka, 2004[362]) who are at high risk of exposure to significant levels of arsenic especially through inhalation (Ham et al., 2017[126]; Park et al., 2010[289]). High levels of urinary arsenic metabolites have been reported in workers from a manufacturing plant (Byun et al., 2013[40]), and were correlated to oxidative injury (Hu et al., 2006[154]). Because of highly contaminated industrial waste effluents from manufacturing plants (Torrance et al., 2010[373]), arsenic threat is not limited to occupational exposure and can affect the surrounding environment through water (Chen, 2006[59]) and air (Chein et al., 2006[55]).

On the other hand, the rapid expansion of technology with rising demand for consumer electronics have resulted in the creation of staggering quantities of electronic waste (e-waste) around the globe. The total e-waste generated worldwide was estimated at approximately 53.6 million tons in 2019, where the contribution of Canada was about 757000 tons (Forti et al., 2020[105]). About 60 chemical elements can be found in various disposed electronics, and some of which is hazardous such as arsenic (Heacock et al., 2016[140]; Yao et al., 2008[428]). The environmental threats of e-waste necessitate efficient recycling, however, in 2019, only 17.4 % of it was officially documented as properly collected and recycled (Forti et al., 2020[105]). Additionally, improper handling of such waste through informal recycling can aggravate the situation and increase the release of toxic substances (Ackah, 2019[1]). In Canada, several organizations are currently working on e-waste recycling through collection, dismantling, hazardous material removal, and recovering of valuable elements (Kumar and Holuszko, 2016[204]; Kumar et al., 2019[205]).

Another industrial application of arsenic is alloying with lead in the manufacturing of lead-acid batteries, which are mostly used as car batteries. Secondary lead smelters produce lead by recovering it from lead-bearing scrap materials (most of which are scrap automobile batteries). Arsenic, among other metals, is typically detected in the area surrounding the recycling facilities (Chai et al., 2015[47]; Eckel et al., 2002[92]; Ettler et al., 2010[98]). Interestingly, arsenic was detected in shed deciduous teeth of children who are living near a lead-acid battery smelter (Johnston et al., 2019[176]). An early study in southern Ontario, has reported high levels of arsenic contamination in soil and vegetation from different locations in the vicinity of two secondary lead smelters (Temple et al., 1977[365]).

#### 3.2.4. Wood preservatives

Arsenic-based wood preservatives, such as chromated copper arsenate (CCA), were developed to prevent its deterioration, especially when intended for outdoor use, by microorganisms or insects. The preservative is applied by pressure treatment and, typically, 1 m^3^ of CCA-treated wood contains about 1.41 kg of arsenic (Morrell and Huffman, 2004[266]). From CCA-treated wood, arsenic can leach through weathering during normal use (Khan et al., 2006[189]), or through disposal via landfilling (Khan et al., 2006[188]; Moghaddam and Mulligan, 2008[260]) or incineration (Wasson et al., 2005[402]).

Zagury et al. have reported high arsenic concentrations in samples from the soil adjacent to the CCA-treated utility poles in Montréal (Québec) (Zagury et al., 2003[437]). Similarly, significant levels of arsenic leaching from CCA-treated utility poles have been detected in western Newfoundland and Labrador (Coles et al., 2014[65]). In Edmonton (Alberta), the average arsenic level on the hands of children playing in playgrounds with CCA-treated wood structures (0.5 μg) was significantly higher than that from playgrounds not constructed with CCA-treated wood (0.095 μg) (Kwon et al., 2004[208]). Of note, the maximum amount of arsenic detected on children hands in that study (< 4 μg) was lower than the reported children average daily intake of total arsenic from food in Canada (14.9 μg) (Health Canada, 2006[141]). As of December 31, 2003, CCA was phased out of residential applications in Canada, and its use is currently restricted to industrial wood products (Wang and Mulligan, 2006[399]).

#### 3.2.5. Pesticides

The inherent toxicity of arsenic has led to its use in wood preservatives as well as agricultural pesticides. Both organic and inorganic arsenic-based compounds were developed and used as insecticides, rodenticides, and herbicides (Bencko and Yan Li Foong, 2017[20]). Arsenical pesticides have a negative impact on the cultivated plants (Quazi et al., 2011[310]), groundwater and surface water (Li et al., 2016[221]; Whitmore et al., 2008[410]), as well as applicators and farmers (Boulanger et al., 2019[33]; Dennis et al., 2010[81]). Additionally, arsenic contamination, because of spills and releases, has been also reported at manufacturing sites (Cancès et al., 2005[42]; Keimowitz et al., 2005[184]). The threat of arsenic-bearing pesticides still exists despite being banned and phased out because of environmental persistence of arsenic residues that resulted from extensive long-term application of these pesticides (Hughes et al., 2011[160]; Quazi et al., 2010[311]).

In southern Ontario, using lead arsenate in apple orchards for over 70 years resulted in more than 10 folds elevation (from 7.4 ppm to 121 ppm) in arsenic level in soil samples (Frank et al., 1976[109]). Similar observations were reported in Annapolis Valley apple orchards (Nova Scotia) (Bishop and Chisholm, 1962[27]). In addition to high arsenic concentrations in soil samples, significant levels were reported in plant tissue from apple orchards and potato fields in the same province (MacLean and Langille, 1981[232]). In Niagara (Ontario), elevated arsenic was detected in samples from trees of different fruits, that had received repeated applications of lead arsenate (Martin et al., 2000[241]).

#### 3.2.6. Feed additives

In animal husbandry, especially poultry, phenylarsonic compounds, most notably of which are roxarsone and nitarsone, have been used as feed additives for improving feed efficiency and protection against parasitic infections. These compounds were originally approved on the basis of being harmless organoarsenicals, however, it has been found that they get converted into inorganic arsenic within the chicken (Nachman et al., 2013[268], 2017[269]). Consequently, their U.S. Food and Drug Administration (FDA) approvals were withdrawn (Chen et al., 2019[60]). The presence of these compounds in poultry litter, which is commonly used as an organic fertilizer, results in soil contamination, where they can undergo biotic (Cortinas et al., 2006[67]; Garbarino et al., 2003[113]; Han et al., 2017[128]) or abiotic (Bednar et al., 2003[17]) conversion into more toxic inorganic species. Eventually, arsenic in the soil may end up in ground water (Rutherford et al., 2003[320]) or the cultivated plants (Huang et al., 2014[156]; Yao et al., 2016[429]).

#### 3.2.7. Drugs

Arsenic is regarded as a double-edged sword, which, despite its toxic nature, has proven therapeutic benefits that date back to the days of Hippocrates who used arsenic sulfides (realgar and orpiment) to treat ulcers and abscesses. Arsenic-based pharmaceuticals have been employed in various disorders throughout history (Henke, 2009[146]), however, a detailed description of these agents started in late 18^th^ century, by the discovery of Thomas Fowler's solution (1 % potassium arsenite solution formed by dissolving As_2_O_3_ in potassium bicarbonate) that was used for a variety of systemic illnesses. In 1878, it was first reported that Fowler's solution can lower the white blood cell counts in leukemia patients, and subsequently, it became the mainstay for the treatment of chronic myelogenous leukemia (CML) until the advent of, the safer, radiation and chemotherapy by the beginning of the 20^th^ century (Waxman and Anderson, 2001[405]). 

In early 20^th^ century, the sodium salt of arsanilic acid, a compound that was discovered 40 years earlier by reacting arsenic acid with aniline, was introduced as the first organoarsenical medicine. This compound was found to be 40 times less toxic than the inorganic Fowler's solution, hence named atoxyl, and was used for the treatment of trypanosomiasis (Riethmiller, 2005[316]). Additional experimentations on atoxyl led Paul Ehrlich, the founder of chemotherapy, to the discovery of arsphenamine, marketed as salvarsan, in 1910. Salvarsan was the “magic bullet” for treating syphilis. Generally, the clinical applications of arsenicals gradually declined because of posing greater health threats than the diseases that they were supposedly curing. Eventually, arsenic medicines have been largely replaced by less toxic compounds. For instance, salvarsan was replaced by penicillin for syphilis treatment (Bosch and Rosich, 2008[32]).

However, some arsenicals are still used, despite their severe toxicity, for treating diseases that typically result in death if untreated, such as the antitrypanosomal melarsoprol (atoxyl was the first effective treatment but blindness was a serious side effect) (Büscher et al., 2017[39]; Steverding, 2010[351]).

The rebirth of As_2_O_3_ therapy occurred in the 1970s as a treatment for acute promyelocytic leukemia (APL), and in 2000, it was approved by FDA as a frontline therapy for this disease (Hoonjan et al., 2018[153]). Because of its success in APL, As_2_O_3_ is currently being investigated for the treatment of other types of cancer (Ally et al., 2016[4]; Huang and Zeng, 2019[158]; Sadaf et al., 2018[322]; Wu et al., 2018[418]).

## 4. Routes of Human Exposure to Arsenic

Humans are exposed to arsenic via several pathways including ingestion of food, drinking water, inhalation of air, or dermal contact (Figure 3(Fig. 3)). Arsenic exposure is a multifactorial process depending on local geochemistry (i.e. natural presence), environmental pollution, and lifestyles of the population. For instance, occupational exposure in industrial environments occurs primarily through inhalation (Xue et al., 2010[424]).

### 4.1. Drinking water

For general population, exposure is mostly oral via ingesting arsenic-contaminated food or water. Drinking water is widely regarded as the major source of exposure especially in areas with arsenic concentrations exceeding the World Health Organization (WHO) guidelines value (10 μg/L) e.g. by living near either a natural geological source or a contaminated site (Cubadda et al., 2017[70]). However, in the presence of water with safe arsenic levels below that limit, food may become a greater contributor to total arsenic intake than drinking water. Assessment of health risks is based on a general understanding that inorganic forms of arsenic are more harmful than organic ones, and that most cases of arsenic-induced toxicity in humans are associated with inorganic arsenic exposure. There is no evidence for the demethylation of organoarsenicals into inorganic forms in mammals (Elshenawy and El-Kadi, 2015[96]). Since aqueous arsenic species are almost exclusively inorganic, compared to only 10 % iAs in food, drinking water is usually considered the greatest menace to human health (Xue et al., 2010[424]).

Various sources of drinking water fall into two main categories; surface water and groundwater. Risk of arsenic exposure may vary depending on the source of water. In anthropogenically impacted areas, all water sources, especially surface water, become vulnerable to contamination. However, naturally, groundwater usually poses higher risk for exposure (Smedley and Kinniburgh, 2002[344]). Extremely high arsenic levels in groundwater may result from its presence at depths where it is exposed to more naturally occurring arsenic sediments. Moreover, drinking water supplied from groundwater is extracted by pumping wells, and such pumping activity causes disruption of soil sediments and facilitates arsenic mobilization to the groundwater (Raessler, 2018[312]). Additionally, groundwater from wells is often not treated before human consumption, because it is generally less accessible to treatment methods than surface water, and its treatment is usually more difficult and expensive (Greco et al., 2019[123]).

In 1980s, the Canadian drinking water guidelines recommended a maximum acceptable concentration (MAC) for arsenic of 50 μg/L (Meranger et al., 1984[249]). However, with the growing knowledge about arsenic-mediated harmful effects as well as the development of more sensitive laboratory methods for detection, that limit was later changed to 25 μg/L (Thirunavukkarasu et al., 2002[366]), and currently a limit matching the published WHO guidelines (10 μg/L) is set by Health Canada (Hu et al., 2020[155]). It is worth mentioning that this limit doesn't warrant protection against arsenic harm (Saint-Jacques et al., 2018[323]), and a limit of 0.3 μg/L would be ideal for achieving an “essentially negligible” lifetime risk of cancer, but 10 μg/L is the lowest concentration that is technically achievable in the Canadian drinking water systems. Generally, arsenic levels in drinking water are less than 5 μg/L in most locations across Canada (Health Canada, 2006[141]).

**Figure 4:**
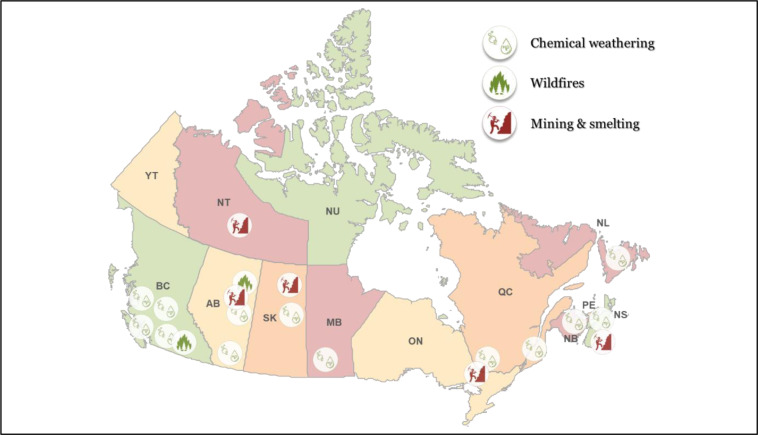
Map of Canada showing notable examples of arsenic sources in different provinces and territories. Natural weathering in specific areas has resulted in hotspots (> 10 μg/L arsenic in water) for arsenic exposure in drinking water. Wildfires such as; 2003 wildfires (Okanagan Mountain Park, BC) and 2016 wildfires (Fort McMurray, AB). Mining & smelting operations in Athabasca oil sands (AB), Giant Mine (Yellowknife, NT), Uranium mine (Rabbit Lake, SK), Silver & cobalt mines (Cobalt town, ON), and Gold mines (NS). *Abbreviations:* AB: Alberta, BC: British Columbia, MB: Manitoba, NB: New Brunswick, NL: Newfoundland and Labrador, NS: Nova Scotia, ON: Ontario, PE: Prince Edward Island, QC: Québec, SK: Saskatchewan, NT: Northwest Territories, NU: Nunavut, YT: Yukon.

However, the natural occurrence of arsenic at high levels in certain locations (Moncur et al., 2015[262]) has created “hotspots” for arsenic exposure through drinking water beyond 10 μg/L (Figure 4(Fig. 4)). This may be a major concern especially in provinces and territories that depend partially (as Alberta) or completely (as Prince Edward Island) on ground water which represent more than 30 % of the population (McGuigan et al., 2010[246]). It would be safer to rely on the controlled municipal drinking water supplies, but approximately 4 million Canadians obtain their water as groundwater through privately-owned domestic wells (Kreutzwiser et al., 2011[199]), and in such case, water is not subject to regulated testing and therefore may contain unknown and possibly unsafe arsenic concentrations as reported in several studies (Chappells et al., 2014[53]; Dummer et al., 2015[90]; Gagnon et al., 2016[111]; Pratt et al., 2016[305]).

### 4.2. Food

Food is another major source of both organic and inorganic arsenic for typical individuals. Arsenic can be found in most diets with varying amounts and forms, i.e. organic or inorganic, depending on the type of food (Uneyama et al., 2007[378]). For instance, seafood represents about 90 % of dietary arsenic exposure in the U.S., of which the vast majority is in complex organic forms. As mentioned earlier, arsenobetaine is the predominant species in marine food, which was found to be not cytotoxic, mutagenic, immunotoxic, or embryotoxic (Borak and Hosgood, 2007[31]; Mania et al., 2015[236]). Comprehensive lists showing the levels of different arsenic species in various food products from different countries can be found in a number of good review articles (Lynch et al., 2014[230]; Upadhyay et al., 2019[379]).

Livestock are exposed to arsenic in contaminated environment through water, plants, incidental soil ingestion, or feed additives. Eventually, inevitable human exposure to arsenic can occur via consuming such animal food products. Studies have reported arsenic exposure through different types of meat (Nigra et al., 2017[278]; Ruiz-de-Cenzano et al., 2017[319]) as well as milk (Datta et al., 2012[77]) and eggs (Ghosh et al., 2012[115]). Because of their arsenic methylation ability, organoarsenicals are the main form in animal food products besides an inorganic fraction (Liu et al., 2016[222]; Nachman et al., 2013[268], 2017[269]). Interestingly, arsenic excreted in milk was found to be entirely inorganic (Datta et al., 2010[76]).

In addition to water, plants are regarded as an important gate for arsenic entry to the food chain when cultivated in arsenic-rich soil or irrigated with arsenic-contaminated water. Therefore, plants are mostly exposed to inorganic forms of arsenic (Huang et al., 2011[156]). Arsenic is considered non-essential for plants and it has no specific uptake system, therefore, it relies on adventitious uptake pathways via various transporters that are naturally intended for minerals and nutrients. For example, iAs^V^ is quite similar to inorganic phosphate (Pi) and can compete with it for the uptake via phosphate transporters. Similarly, the uptake of iAs^III^, which is the dominant species in anaerobic environments, can be achieved by silicon (Si) transporters due to structural similarity between arsenious acid and silicic acid which both exist as neutral species in such environments (Zhao et al., 2009[443]). Because of the competition of iAs^V^ and iAs^III^ with these structurally similar species, plants can be protected from arsenic by using phosphate and silicon supplements, respectively (Kumarathilaka et al., 2020[206]).

In contaminated environments, the overwhelmingly high arsenic concentrations result in extensive uptake and accumulation in their edible parts. Additionally, while animals can metabolize and excrete excess iAs resulting in low iAs quantities in their food products (Cubadda et al., 2017[70]), higher plants have no methylation ability for iAs because of lacking the required genes (Tang et al., 2016[363]). Therefore, consumption of plant-derived food products, such as fruits and vegetables, may result in exposure to high levels of iAs (Cubadda et al., 2017[70]).

Rice is one of the most severely arsenic-affected plants because of its special cultivation method in flooded paddy soils that creates an ideal anaerobic environment for iAs^III^. Since it requires large amounts of Si for its optimal growth, rice is a very efficient plant in accumulating Si (making up to 10 % of the shoot biomass) (Chen et al., 2017[61]). Subsequently, excessive inadvertent uptake of iAs^III^ takes place, which is then translocated to rice grains resulting in about 10 folds of the iAs accumulated in other grains such as wheat and barley (Davis et al., 2017[78]). The fact that rice is a globally important food crop and a primary daily source of calories for more than half the world's population, renders it a potential source of human exposure to iAs (Khush, 2005[190]). Additionally, high concentrations of iAs can be also found in rice-based products including baby rice, rice cereals and rice crackers consumed by infants and young children who are especially vulnerable to the adverse health effects (Jackson et al., 2012[169]; Signes-Pastor et al., 2016[340]).

In addition to food products, the presence of arsenic in other plant-based products such as tobacco leaves imply a significant exposure through cigarette smoke (Mierzwa et al., 1997[258]; Taebunpakul et al., 2011[359]). Arsenic has been found to act synergistically with other carcinogens in cigarette smoke in the induction of lung cancer (Hertz-Picciotto et al., 1992[150]).

### 4.3. Air

A relatively much lower arsenic exposure can result from inhalation of polluted air in which arsenic is mostly present in an inorganic form adsorbed onto particulate matter. This kind of exposure is commonly related to emissions in industrial environments where significant arsenic levels are released to the atmosphere (Meacher et al., 2002[247]). Exposure to volatile arsines may happen especially in the vicinity of their, previously mentioned, releasing sources (Lewis et al., 2012[220]).

In remote areas away from anthropogenic releases, the average atmospheric level of arsenic is 0.02-4 ng/m^3^, while in urban areas may reach 200 ng/m^3^. Concentrations of several hundred nanograms per cubic meter have been reported in some cities especially in industrially impacted areas (IARC, 2012[162]). In Canada, a significant decline in the levels of major air pollutants, including arsenic, have been observed over the past four decades (IARC, 2016[163]). The mean airborne concentration of arsenic in 11 Canadian cities and one rural site monitored from 1985 to 1990 was 0.001 µg/m^3^ (CEPA, 1993[46]). According to the Canadian National Air Pollution Surveillance (NAPS) monitoring system, the average concentration of arsenic measured in outdoor air in 2011 was 0.00043 μg/m^3^ (Setton et al., 2013[329]). Much higher arsenic concentrations have been recorded in industrial zones (Wang and Mulligan, 2006[399]).

### 4.4. Dermal exposure

Dermal contact is another route of arsenic exposure associated with relatively low risk of poisoning. Exposure may happen through water (Ouypornkochagorn and Feldmann, 2010[286]; Smith et al., 2016[345]), soil (Lowney et al., 2007[225]), and arsenic-preserved wood structures (Chen and Olsen, 2016[56]; Hemond and Solo-Gabriele, 2004[145]). Individuals suffering from blackfoot disease, a severe vascular disease associated with long-term arsenic exposure via drinking water, usually have concurrent occupational dermal exposure to arsenic-contaminated water and soil through farming, fishery, or salt production (Irfan, 2012[165]; Tseng, 2005[374]). Arsenic in soil occurs primarily in inorganic forms (Wang and Mulligan, 2006[399]), and, besides dermal exposure, incidental ingestion can be a significant exposure pathway for soil especially among children while playing (Bacigalupo and Hale, 2012[12]; Ljung et al., 2006[224]).

## 5. Modulation of CYP Enzymatic Machinery by Arsenic

### 5.1. CYPs as a key player in metabolic biotransformation

Metabolic biotransformation in biological systems aims at maintaining physiological homeostasis by generating energy and building functional and structural molecules (such as proteins and lipids) from consumed food, as well as eliminating catabolic wastes. This process comprises a wide range of enzyme-catalyzed reactions arranged in well-defined metabolic pathways in which a substrate is sequentially converted to the desired end-product. Human body may encounter a non-nutritious foreign substance that is not expected to be naturally present within the system (such as environmental pollutants and drugs), namely a xenobiotic. In this case, the metabolic machinery acts as a defense system that attempts to detoxify the foreign compound by modifying its chemical structure to deactivate it and facilitate its excretion. However, sometimes, xenobiotic metabolism backfires by producing more active intermediates with subsequent detrimental effects. In mammals, different organs (such as lung, kidney, heart, brain, skin, and intestine) contribute to metabolism (including xenobiotic biotransformation) in the body; however, the liver is considered the major contributor through its diverse arsenal of enzymes (De Kanter et al., 2002[79]).

CYPs constitute a superfamily of heme-containing monooxygenase enzymes, a part of which represents a major class of xenobiotic-metabolizing enzymes involved in the oxidative biotransformation of most drugs and other lipophilic xenobiotics (Guengerich, 2008[124]). These enzymes are ubiquitous and have been identified in all kingdoms of life (Lamb et al., 2009[213]). CYPs are prominent metabolic enzymes that are found primarily in hepatic microsomes in addition to other extrahepatic tissues (Ding and Kaminsky, 2003[84]). In humans, there are 57 members in CYP superfamily that are grouped into 18 families and 44 subfamilies based on their sequence homology. Most of these enzymes have specific endogenous metabolic functions including the metabolism of fatty acids (such as arachidonic acid), cholesterol, bile-acids, steroid hormones, vitamin D, and others (Nebert and Russell, 2002[276]). Being physiologically involved in metabolizing endogenous substrates, derangements in CYPs function have been implicated in several disease states. In this case, CYPs can be reversely exploited as targets for treating such pathological conditions (Navarro-Mabarak et al., 2018[273]; Wang et al., 2019[400]; Xu et al., 2011[423]).

Besides endogenous substrates, members belonging to the CYP1, CYP2, and CYP3 families are collectively involved in the xenobiotic metabolism of the majority of drugs and other foreign chemicals (Zanger and Schwab, 2013[439]). For instance, it is estimated that about 75 % of marketed drugs undergo CYP-mediated hepatic elimination, mostly through metabolic pathways involving CYP3A4/5, CYP2C9, CYP2D6, CYP2C19, and CYP1A2 (Guengerich, 2008[124]; Zanger et al., 2008[440]). Because of such deep involvement in xenobiotic biotransformation, CYPs can significantly modulate the overall body exposure to foreign chemicals through either detoxification or bioactivation. Therefore, CYPs mediating such biotransformation have been widely studied for their toxicological implications (Guengerich, 2008[124]).

CYPs activity may reduce the efficacy and/or toxicity of a drug by accelerating the elimination of its active form. In other circumstances, such metabolic activity may enhance the efficacy or toxicity of a drug by activating its inert prodrug or generating toxic metabolites, respectively (McDonnell and Dang, 2013[245]). Consequently, alteration of CYPs activity in relation to certain drug can result in crucial modification in its behavior inside the body and the ultimate outcome of its exposure. That is why induction or inhibition of CYPs by concomitant medications can result in clinically relevant drug interactions (Storelli et al., 2018[353]), that may necessitate revising and updating safety profiles of pharmaceutical products (Yoshida et al., 2006[434]).

The impact of CYPs activity, and the possible alteration of such activity, is not limited to drugs but extends to include all foreign chemicals undergoing CYPs-mediated biotransformation that may be altered by co-exposure to other xenobiotics capable of modulating CYPs metabolizing activity. Environmental contaminants form a major cluster of xenobiotics that are hazardous to humans. Additionally, they may accumulate in the environment, due to their recalcitrant properties and long degradation periods, thus aggravating their threat to human health (Manzetti, 2013[237]). Polycyclic aromatic hydrocarbons (PAHs) represent a notable family of these pollutants, which are well-known for their toxic and carcinogenic properties. These compounds are mainly produced in the environment as airborne contaminants resulting from incomplete combustion of organic matter such as fossil fuels (Kim et al., 2013[191]). Benzo[a]pyrene (B[a]P) is a widely studied member of this family which is a potent lung carcinogen found at high levels in cigarette smoke (Hecht, 1999[144]). B[a]P is a procarcinogen whose bioactivation into a mutagenic intermediate is based on its capacity to stimulate its own metabolism. As a PAH, B[a]P induces the production of its metabolizing enzymes, most notably CYP1A1, via activating its master regulator; the aryl hydrocarbon receptor (AhR). Through its diol epoxide metabolite, B[a]P form covalent DNA adducts by interacting with N^2^-position of guanine in critical genes such as the p53 tumor suppressor, as commonly seen in lung cancer smokers, resulting in initiation of tumorigenesis (Badal and Delgoda, 2014[13]; Shimada et al., 2002[337]).

In this case, cancer risk evaluation might be underestimated if based only on the sole exposure to such CYPs-dependent carcinogen, because human body is exposed daily to various pollutants and co-exposure to complex mixtures of contaminants is inevitable. These co-contaminants can enhance the bioactivation of other contaminants through manipulating their activating enzymes. These co-contaminants include heavy metals such as arsenic (Anwar-Mohamed et al., 2009[6]). Several epidemiological studies have reported significantly high incidence of lung cancer among cigarette smokers who are concurrently exposed to arsenic (Chen et al., 2004[58]; Ferreccio et al., 2000[102]; Hertz-Picciotto and Smith, 1993[149]; Hertz-Picciotto et al., 1992[150]; Järup and Pershagen, 1991[173]; Pershagen et al., 1981[294]; Tsuda et al., 1995[375]). Studies on animals have also revealed that tumorigenic potential of B[a]P in the respiratory tract can be significantly enhanced by arsenic co-exposure (Ishinishi et al., 1977[166]; Pershagen et al., 1984[293]). Considering that arsenic is a well-established carcinogen (Wei et al., 2019[406]), the potentiated B[a]P effect may be regarded as synergistic co-carcinogenesis caused by both of them as shown by rat lung cell transformation rate that has increased beyond 500- and 200-folds compared with arsenic alone or B[a]P alone, respectively (Lau and Chiu, 2006[216]). It has been also reported that arsenic enhances the benzo[a]pyrene diol epoxide (BPDE)-DNA adduct-induced mutagenesis in the lung (Chiang and Tsou, 2009[62]; Evans et al., 2004[99]). Interestingly, CYP1A1, the key activator of B[a]P, was found to be induced in the lung by arsenic exposure at the levels of mRNA, protein, and/or catalytic activity in both *in vivo *and* in vitro* studies (Albores et al., 1995[3]; Anwar-Mohamed et al., 2012[5]; Cameron Falkner et al., 1993[41]; Elshenawy et al., 2018[94]; Elshenawy and El-Kadi, 2015[96]; Seubert et al., 2002[330]; Wu et al., 2008[419]). Although some studies do not support this effect (Cameron Falkner et al., 1993[41]; Elshenawy et al., 2018[94]; Ho and Lee, 2002[151]; Seubert et al., 2002[331]), arsenic-mediated positive modulation of CYP1A1 remains a potential clue to the high incidence of lung cancer among cigarette smokers. 

### 5.2. Transcriptional regulation of CYPs expression

The expression of different CYP isoforms is subject to the control of an intricate network of various transcription factors (Omiecinski et al., 2011[284]; Zanger and Schwab, 2013[439]), and here we focus on two key regulators involved in arsenic studies, the AhR and the pregnane X receptor (PXR), because of the significant clinical impacts of their associated CYP enzymes.

**Figure 5:**
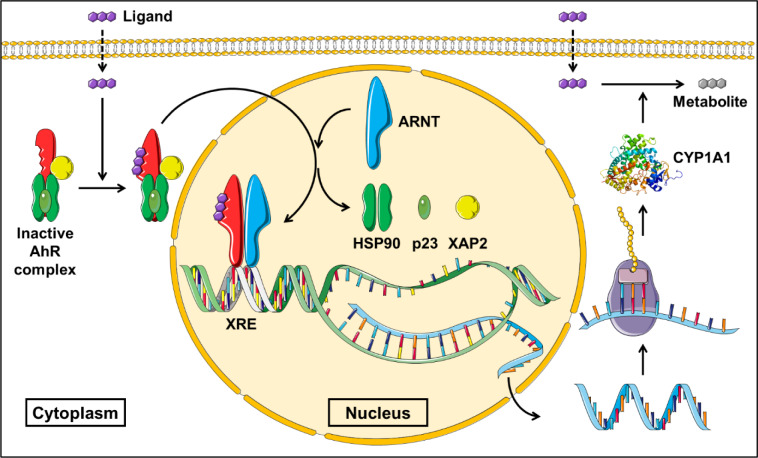
AhR signaling pathway. The unliganded AhR resides in the cytoplasm, complexed with a dimer of the chaperone heat shock protein 90 (HSP90), the co-chaperone prostaglandin E synthase 3 (p23), and a molecule of hepatitis B Virus X-associated protein 2 (XAP-2). Ligand-mediated activation of the AhR results in its nuclear translocation where it dissociates from its complex and forms a heterodimer with the aryl hydrocarbon receptor nuclear translocator (ARNT) that binds to the xenobiotic response element (XRE) found in the promoter regions of AhR-regulated genes such as CYP1A1.

AhR is a ligand-activated bHLH/Per-ARNT-Sim transcription factor which is retained in the cytoplasm as an inactive complex with a dimer of the chaperone heat shock protein 90 (HSP90), the co-chaperone prostaglandin E synthase 3 (p23), and a molecule of hepatitis B Virus X-associated protein 2 (XAP-2) (Figure 5(Fig. 5)). Upon binding to one of its agonists, such as PAHs or halogenated aromatic hydrocarbons (HAHs), AhR molecule undergoes a conformational change exposing its nuclear localization sequence (NLS). Eventually, the activated AhR translocates into the nucleus where it dissociates from its cytoplasmic complex and dimerizes with the aryl hydrocarbon receptor nuclear translocator (ARNT) to form a heterodimer that binds to the xenobiotic response element (XRE), also known as dioxin response element (DRE), found in the promoter regions of AhR-regulated genes (Beischlag et al., 2008[18]; Larigot et al., 2018[214]; Soshilov and Denison, 2008[346]).

The name of AhR was originally based on the assumption that it functions primarily as a sensor for xenobiotic chemicals, the most notable of which are aromatic (aryl) hydrocarbons such as PAHs (e.g. benzo[a]pyrene, 3-methylcholanthrene, and beta-naphthoflavone) and HAHs (e.g. 2,3,7,8-Tetrachlorodibenzo-p-dioxin). However, extensive studying of the AhR has revealed its promiscuous ligand specificity that allows binding to a large number of structurally diverse chemicals. Besides PAHs and HAHs, some natural exogenous compounds such as flavonoids (e.g. quercetin, kaempferol (Ciolino et al., 1999[63]), and resveratrol (Casper et al., 1999[45])) and indoles (e.g. indole-3-carbinol (Hammerschmidt-Kamper et al., 2017[127])) have been found to act as AhR ligands. Additionally, several endogenously formed molecules have been identified as AhR ligands, such as the indole amino acid (tryptophan) and its catabolites (e.g. tryptamine, indole acetic acid (Heath-Pagliuso et al., 1998[143]; Hubbard et al., 2015[159]), and kynurenic acid (DiNatale et al., 2010[83])), as well as other indoles (e.g. indirubin and indigo) (Adachi et al., 2001[2]). Other endogenous ligands include tetrapyrroles (e.g. bilirubin (Sinal and Bend, 1997[342]) and biliverdin (Phelan et al., 1998[298])) and arachidonic acid metabolites (e.g. lipoxin A4 (Schaldach et al., 1999[326]) and some prostaglandins (Seidel et al., 2001[327])).

AhR is a gene battery that regulates a group of phase I as well as phase II enzymes (Anwar-Mohamed et al., 2009[6]). The expression of CYP1A1, CYP1A2, CYP1B1, and CYP2S1 genes, which represent phase I group, is regulated by AhR response elements found in their promoters, therefore they are highly inducible by AhR ligands (Jorge-Nebert et al., 2010[179]; Kerzee and Ramos, 2001[185]; Saarikoski et al., 2005[321]; Ueda et al., 2006[377]). In humans, CYP1A1, CYP1A2, and CYP1B1 are constitutively expressed in the liver; however, only CYP1A2 is detected at much higher levels. CYP1A1 and CYP1B1 are primarily extrahepatic enzymes and their hepatic levels are very low or undetectable (Zanger and Schwab, 2013[439]). Human CYP2S1 levels are generally low across the different organs in the body (including the liver) (Deb and Bandiera, 2009[80]). AhR-regulated CYPs 1A1, 1A2, and 1B1 have gained significant attention because of their ability to activate the procarcinogenic AhR ligands (Shimada and Fujii-Kuriyama, 2004[335]; Shimada and Guengerich, 2006[336]).

Another ligand-activated transcription factor is the PXR, also known as steroid and xenobiotic receptor (SXR) or NR1I2, which is a member of the nuclear receptor (NR) superfamily. Mouse PXR (mPXR) cytosolic localization is maintained through its engagement in a multiprotein complex, composed of cytoplasmic CAR retention protein (CCRP) and HSP90, which gets disassembled through ligand-mediated activation, thus allowing the receptor to translocate into the nucleus (Squires et al., 2004[349]; van de Winkel et al., 2011[393]). Alternatively, human PXR (hPXR) has been reported to be a predominantly nuclear protein regardless of ligand binding or activation status (Kawana et al., 2003[183]; Koyano et al., 2004[198]; Saradhi et al., 2005[325]). Ultimately, the activated PXR forms a heterodimer with another nuclear receptor, namely, the retinoid X receptor alpha (RXRα), also known as NR2B1, which binds to the PXR response module located in the promoter regions of its target genes (Carnahan and Redinbo, 2005[44]).

PXR name was coined by Kliewer et al. after observing that the receptor was activated by both natural (e.g. pregnenolone and progesterone) and synthetic (e.g. dexamethasone and pregnenolone 16α-carbonitrile) pregnane (21-carbon) steroids (Kliewer et al., 1998[195]). The human PXR was initially reported as the steroid and xenobiotic receptor (SXR) by Blumberg et al. because of its ability to interact with natural steroids as well as xenobiotic drugs (including synthetic steroids) (Blumberg et al., 1998[28]). PXR can recognize and accommodate a wide range of structurally diverse endogenous and exogenous ligands, and this may be attributed to its large and flexible ligand-binding pocket (Watkins et al., 2001[404]). A myriad of natural (endobiotics and xenobiotics) and synthetic compounds have been shown to bind with PXR including steroids (e.g. pregnanes, estranes, androstanes (Blumberg et al., 1998[28]; Kliewer et al., 1998[195]), and bile acid precursors (Goodwin et al., 2003[119])), clinically used drugs (e.g. rifampicin and nifedipine) (Honkakoski et al., 2003[152]; Kliewer et al., 2002[194]; Shukla et al., 2011[339]), herbal compounds (e.g. hyperforin; a constituent of St. John's Wort) (Chang, 2009[51]; Staudinger et al., 2006[350]), and environmental contaminants (e.g. organobromine (Pacyniak et al., 2007[287]) and organochlorine (Coumoul et al., 2002[69]) compounds).

Activation of PXR is associated with a broad range of transcriptional targets including both phase I and phase II enzymes as well as phase III transporters (Iyer et al., 2006[168]; Tolson and Wang, 2010[372]). A primary target of PXR activation is the induction of CYP3A4 (Istrate et al., 2010[167]), in addition to other CYPs such as CYP3A5 (Burk et al., 2004[37]), CYP3A7 (Burk et al., 2002[38]), CYP2B6, CYP2C9 (Drocourt et al., 2001[89]; Goodwin et al., 2001[120]), and CYP4F12 (Hariparsad et al., 2009[135]).

CYP3A subfamily members are constitutively expressed in various tissues especially in the liver and intestine where they, especially CYP3A4, represent the predominant CYPs. This subfamily has gained its importance from its contribution to both first-pass and systemic metabolism of more than 50 % of the clinically used drugs, thus dictating their therapeutic outcome, along with many other xenobiotics and endobiotics (Martignoni et al., 2006[240]; Woodland et al., 2008[416]). CYP3A enzymes expression is mainly regulated by PXR whose activation is predictive of their induction. That is why PXR has been significantly detected in the same tissues of high CYP3A expression and it has been found to be activated by established CYP3A inducers. Interestingly, PXR assays are implemented in pharmaceutical industry for identification and elimination of CYP3A-inducing candidates at early stages of drug discovery because of the high potential of drug interactions (Goodwin et al., 2002[121]; Kliewer, 2015[193]; Kliewer et al., 2002[194]; LeCluyse, 2001[217]). CYP3A4 is the most abundant and extensively studied member of this subfamily which is strongly tied to the xenosensor PXR as a notable part of its repertoire of xenobiotic metabolizing enzymes (Lehmann et al., 1998[218]; Martignoni et al., 2006[240]; Zhou et al., 2009[444]).

The contribution of PXR-regulated CYP3A enzymes to the clearance of a wide range of xenobiotics, thus diminishing their toxicity, has provided a solid explanation of steroidal catatoxic effect. The concept of “catatoxic steroids”, first introduced by Hans Selye (Selye, 1969[328]), describes the ability of natural and synthetic steroids to confer resistance against (the Greek cata = down, against) various xenobiotics and their harmful effects (Blumberg et al., 1998[28]). The molecular basis of such activity was later attributed to two parallel components including the activation of PXR by structurally diverse ligands, and subsequent upregulation of detoxifying enzymes with broad substrate specificity; the CYP3A subfamily (Kliewer, 2015[193]; Kliewer et al., 1998[195]).

PXR DNA-binding domain (DBD) in different species is almost 95 % identical, however, uncommonly in nuclear receptors, PXR shows explicit cross-species variation attributed to differences in amino acid sequences of its ligand binding domain (LBD) across mammalian species. For example; rabbit, rodent, and human PXR share only about 80 % amino acid identity in their LBDs, which is strikingly lower than what is typically exhibited by orthologous nuclear receptors (Iyer et al., 2006[168]; Jones et al., 2000[178]). Subsequently, substantially divergent PXR activation profiles are observed among these species. For instance, rifampicin efficiently activates human and rabbit PXRs with almost no activity in mouse or rat, while pregnenolone 16α-carbonitrile (PCN) activity on mouse and rat PXRs is much more prominent compared with that on rabbit and human receptors. Such inter-species variability is mirrored into species-specific CYP3A induction pattern. Similarly, rifampicin induces human and rabbit but not rodent CYP3A, while PCN induces rodent CYP3A with little effect on human or rabbit CYP3A (Carnahan and Redinbo, 2005[44]; Kliewer et al., 2002[194]; LeCluyse, 2001[217]; Östberg et al., 2002[285]; Quattrochi and Guzelian, 2001[309]).

This species‐specific PXR behavior leads ultimately to markedly different xenobiotic response across species, something that complicates the relevance of experimental animal models to hPXR pharmacology. This has led to the development of a humanized PXR mouse created by introducing hPXR to a PXR-null mouse (mPXR^-/-^), yielding ultimately a “humanized” CYP3A induction profile. The transgenic mouse, which is deficient in mPXR gene while expressing a hPXR transgene, will eventually respond to human, not rodent, PXR activators. Consequently, the activated hPXR, which has almost identical DBD as mPXR, can bind at the promoter of CYP3A thus inducing the mouse orthologue of the human enzyme (Woodland et al., 2008[416]; Zhou et al., 2009[444]). CYP3A11, the mouse orthologue of human CYP3A4 (Martignoni et al., 2006[240]), is induced by rifampicin, but not PCN, in this model (Ma et al., 2007[231]; Xie et al., 2000[421]). Again, this animal model clearly proves the deep involvement of PXR in CYP3A regulation and that inter-species variability in CYP3A expression is attributed to structural variation of PXR. Similarly, this principle has been also applied *in vitro* where the rat orthologue of human CYP3A4, CYP3A23 (Ma et al., 2007[231]; Willson and Kliewer, 2002[413]), was significantly induced by rifampicin in hPXR-transfected rat hepatocytes (Xie et al., 2000[421]).

It is worth mentioning that CYP3A induction has been found to be partly co-regulated by the constitutive androstane receptor (CAR; NR1I3) (Pascussi et al., 2003[291]; Reschly and Krasowski, 2006[315]) in addition to other nuclear receptors including the bile acid receptor/farnesoid X receptor (BAR/FXR; NR1H4) (Gnerre et al., 2004[117]), the glucocorticoid receptor (GR; NR3C1) (Dvorak et al., 2003[91]), and the vitamin D receptor (VDR; NR1I1) (Thummel et al., 2001[368]). The xenosensing nuclear receptors PXR and CAR are closely related with overlapping transcriptional targets. They share common CYP3A response elements, therefore a cross-talk between them is possibly involved in xenobiotic metabolic response (Woodland et al., 2008[416]).

The early *in vitro* transfection assays have revealed the constitutive transcriptional activity of the CAR where spontaneous nuclear accumulation, heterodimerization with the RXRα, and subsequent activation of target gene transcription take place in the absence of a ligand (Qatanani and Moore, 2005[307]; Wang and LeCluyse, 2003[397]). However, screening for potential ligands has identified the androstane metabolites, andostranol and androstanol, as endogenous ligands which bind to activated CAR and act as inverse-agonists. Reversal of the intrinsically high constitutive activity of apo-CAR, i.e. unliganded conformation, by these compounds can minimize the formation of toxic metabolites from some drugs as acetaminophen (Qatanani and Moore, 2005[307]; Shan et al., 2004[332]). In its native environment such as primary hepatocytes or *in vivo*, unlike the heterologous cell types, CAR is sequestered in the cytosol not the nucleus (Shan et al., 2004[332]; Xu et al., 2005[422]). Similar to PXR, the activated CAR is dissociated from its cytoplasmic complex with CCRP and HSP90, then translocates into the nucleus to ultimately dimerize with the RXRα and bind as a heterodimer to its response element in target gene promoter (Timsit and Negishi, 2007[369]; Xu et al., 2005[422]). Through a classical ligand-binding mechanism, CAR activation and nuclear accumulation is triggered by direct binding to agonist ligands that potentiate its constitutive activity. Interestingly, CAR can be also indirectly activated and translocated in a ligand-independent manner which seems to be the predominant mode of its activation (Yang and Wang, 2014[427]). For instance, phenobarbital is a well-known CYP3A inducer whose effect is mediated by indirect CAR activation (Sueyoshi and Negishi, 2001[355]).

### 5.3. Arsenicals-mediated alteration of CYPs expression in human-based experimental models

For years, different arsenic species have been studied for their modulatory effects on different CYPs, and have shown species-, tissue-, and/or enzyme-specific effects. Identifying these effects is highly important in understanding how these compounds affect different tissues in the human body, and this can be exploited in either establishing preventive measures for arsenic toxicity or developing therapeutic strategies for treating certain diseases. Arsenic-mediated alteration of the CYPs has been reported at multiple levels of their expression including mRNA, protein, and catalytic activity. Some studies have also investigated the influence of arsenic on the transcriptional regulators of these enzymes. These CYPs-regulating transcription factors act downstream of signaling cascades related to biological/environmental stimuli.

Experimental animal models represent a major avenue of research especially in the field of toxicology where using human subjects is, obviously, impossible. However, extrapolating experimental data from animals to humans can be very complex and may result in poor prediction of human reactions to different xenobiotics. That is why bridging studies using human *in vitro* models constitute an indispensable tool for elucidating human responses (Wrighton et al., 1995[417]). For instance, difference in metabolic behavior, resulting from species-specific enzyme expression or activity, is a hallmark of inter-species variability in xenobiotic handling that eventually complicates the translation of exposure outcomes in animals to humans (Astashkina et al., 2012[9]). Being at the core of the metabolic system, CYPs are no exception. Species-related disparity in catalytic activity/specificity of some CYP isoforms may produce different induction/inhibition patterns for the enzymes. Additionally, inter-species differences can also originate from varying expression of specific isoforms among species (Martignoni et al., 2006[240]). Accurate prediction of human metabolic response can be achieved by using human-based *in vitro* models, especially for the liver which is the major metabolic organ, such as cellular systems (e.g. primary liver cells and derived cell lines), as well as enzymes preparations (e.g. tissue homogenates, subcellular fractions, and purified enzymes) (Costa et al., 2014[68]; Wrighton et al., 1995[417]; Zhang et al., 2012[441]). Because of the reliable *in vitro-in vivo* correlation provided by these human *in vitro *models, FDA can waive clinical drug-drug interaction studies when a drug candidate is tested negative in human *in vitro* CYP induction studies (Zhang et al., 2012[441]). 

Throughout reviewing the literature, we have come across a plethora of studies investigating arsenic-related effects on different members of CYP superfamily using various animal models, but here we shed light on studies based on human *in vitro* models (Table 1(Tab. 1); References in Table 1: Anwar-Mohamed and El-Kadi, 2010[7]; Anwar-Mohamed et al., 2014[8]; Bessette et al., 2005[23], 2009[24]; Bonzo et al., 2005[30]; Chao et al., 2006[52]; Elshenawy et al., 2017[95]; Ho and Lee, 2002[151]; Mondal et al., 2018[263]; Noreault et al., 2005[280]; Noreault-Conti et al., 2012[281]; Spink et al., 2002[347]; Tully et al., 2000[376]; Vakharia et al., 2001[390][391]; Vernhet et al., 2003[394]; Wu et al., 2003[420], 2008[419]). These studies should, to a great extent, depict what would happen inside the human body upon exposure to this toxicant.

The most commonly used experimental model in these studies was liver cells especially human hepatoma (HepG2) cells and primary human hepatocytes. Out of all arsenic species, the trivalent inorganic arsenite has drawn most attention from researchers who assessed its effect specifically on AhR-regulated CYP1 family as well as PXR-regulated CYP3A4.

In liver cells, inorganic arsenic species and organoarsenicals have opposite effects on CYP1A1 mRNA and protein levels. On one hand; arsenite (Elshenawy et al., 2017[95]) and arsenic trioxide (Vernhet et al., 2003[394]) cause reduction in CYP1A1 mRNA transcripts and protein produced constitutively and/or induced by well-known inducers as TCDD (2,3,7,8-Tetrachlorodibenzo-p-dioxin), B[k]F (Benzo[k]fluoranthene), and 3-MC (3-methylcholanthrene), but on the other hand; monomethylarsonic acid, dimethylarsinic acid, and trimethylarsine oxide (Anwar-Mohamed et al., 2014[8]) cause significant increase at both mRNA and protein levels. Interestingly, monomethylarsonous acid is the only organic species which has effects matching these of arsenite and arsenic trioxide (Elshenawy et al., 2017[95]).

Actinomycin D chase studies assessing CYP1A1 mRNA stability have revealed no effect exerted by either arsenite (Anwar-Mohamed and El-Kadi, 2010[7]) or monomethylarsonous acid (Elshenawy et al., 2017[95]). However, monomethylarsonous acid, but not arsenite, decreases the protein stability of CYP1A1 as shown by cycloheximide chase experiments (Elshenawy et al., 2017[95]).

The effect of the mentioned arsenicals on EROD (7-ethoxyresorufin O-deethylation) activity of CYP1A1 follows the same pattern as what has been observed with mRNA and protein. Additionally, incubation of arsenite with human recombinant CYP1A1 (supersomes) results in a significant decrease in its 17β-estradiol 2-hydroxylation activity (Spink et al., 2002[347]). Also, monomethylarsonous acid has a direct inhibitory effect on EROD activity of TCDD-induced CYP1A1 (Elshenawy et al., 2017[95]).

Arsenite has organ-specific effects on CYP1A1 as shown from studies on the cells derived from extrahepatic tissues. For instance, arsenite potentiates CYP1A1 mRNA basal level in human lung adenocarcinoma (H1355) cells (Wu et al., 2008[419]), but has no effect on its basal or inducible protein levels in human lung adenocarcinoma (CL3) cells (Ho and Lee, 2002[151]). In human breast cancer (T-47D) cells, arsenite doesn't alter B[a]P-induced CYP1A1 mRNA but causes significant reduction in its inducible protein levels as well as 17β-estradiol 2-hydroxylation activity (Spink et al., 2002[347]; Wu et al., 2003[420]).

Because of being subjected to the same transcriptional regulation via AhR, it is not surprising that CYP1A2 is similarly affected by arsenicals as CYP1A1. Arsenite causes significant reduction in inducible CYP1A2 mRNA, protein, as well as EROD (Vakharia et al., 2001[390]) and MROD (7-methoxyresorufin O-demethylation) (Anwar-Mohamed and El-Kadi, 2010[7]) activities. Besides decreasing the inducible level of CYP1A protein, monomethylarsonous acid reduces its stability as well (Elshenawy et al., 2017[95]). CYP1B1 is another AhR-regulated enzyme whose basal protein level, in human breast epithelial (MCF10A) cells (Mondal et al., 2018[263]), and induced 17β-estradiol 4-hydroxylation activity, in T-47D cells (Spink et al., 2002[347]), significantly decrease in response to arsenic trioxide and arsenite treatments, respectively. Also, incubation of arsenite with human recombinant CYP1B1 (supersomes) causes significant reduction in its 17β-estradiol 4-hydroxylation activity (Spink et al., 2002[347]).

The above-mentioned findings about CYP1A1, CYP1A2, and CYP1B1 have been further elucidated by studies investigating their upstream transcriptional control by the AhR. Immunocytochemical analysis of AhR localization have revealed significant reduction in TCDD-stimulated nuclear localization of the AhR in HepG2 cells co-treated with either arsenite or monomethylarsonous acid (Elshenawy et al., 2017[95]). On the other hand, the methylated arsenicals; monomethylarsonic acid, dimethylarsinic acid, and trimethylarsine oxide cause significant increase in AhR nuclear accumulation (Anwar-Mohamed et al., 2014[8]). AhR transcriptional activity has been assessed through luciferase-based reporter assays. HepG2 cells and human hepatoma (Hep3B) cells transfected with reporter constructs, carrying CYP1A1 gene promoter sequence located upstream of the firefly luciferase reporter gene, have shown AhR-dependent induction of firefly luciferase activity (normalized using *Renilla* luciferase activity in a dual-luciferase reporter assay) after being treated with B[k]F and 3-MC, respectively. However, arsenite (Bessette et al., 2005[23]) and arsenic trioxide (Vernhet et al., 2003[394]) significantly decrease B[k]F and 3-MC-induced activity, respectively. Arsenite and monomethylarsonous acid (Elshenawy et al., 2017[95]), but not arsenic trioxide (Vernhet et al., 2003[394]), reduce both basal and inducible AhR-dependent XRE-driven firefly luciferase reporter activity. In case of monomethylarsonic acid, dimethylarsinic acid, and trimethylarsine oxide; an opposite effect on XRE-mediated luciferase activity has been observed in both absence and presence of TCDD (Anwar-Mohamed et al., 2014[8]).

H1355 cells transfected with XRE-luciferase genetic construct have shown significant increase in reporter activity in response to arsenite treatment, i.e. opposing its effect in liver cells (Wu et al., 2008[419]). Also, contrary to what has been observed with inorganic arsenic species, Tully et al. have reported that arsenate causes increase in AhR-dependent reporter signal (Tully et al., 2000[376]). This study used CAT-Tox (L)iver assay system which is a recombinant cell line derived from HepG2 cells and contains either CYP1A1 gene promoter or XRE fused to the chloramphenicol acetyl transferase (CAT) reporter gene (Todd et al., 1995[371]).

Arsenite has been found to be negatively affecting CYP3A4 in primary human hepatocytes at the levels of mRNA, protein, and enzymatic activity. Both constitutively expressed and induced, by either rifampicin or phenobarbital, CYP3A4 mRNA and protein decrease in response to arsenite treatment. CYP3A4 testosterone 6β-hydroxylation is similarly affected by arsenite (Noreault-Conti et al., 2012[281]; Noreault et al., 2005[280]).

PXR, the key regulator of CYP3A4, has not exhibited any alteration in its mRNA or protein in arsenite-treated primary human hepatocytes. However, when primary cultures of rat hepatocytes, prepared from mature male Fisher 344 rats, were co-transfected with a construct of CYP3A4 rat orthologue (CYP3A23) promoter-luciferase reporter as well as a plasmid containing the complete protein-coding region of human PXR, the reporter activity was induced by rifampicin, a known activator of human but not rat PXR (in this case, it acts upon the ectopically expressed human PXR), but such activity was significantly reduced by arsenite treatment (Noreault et al., 2005[280]). Similarly, arsenite decreases rifampicin-induced luciferase activity in HepG2 cells co-transfected with CYP3A23-luciferase reporter and ectopic human PXR (Noreault-Conti et al., 2012[281]). Interestingly, both constitutive and rifampicin-induced RXRα, a transcription factor that regulates CYP3A4 gene transcription as a heterodimer with PXR, mRNA and protein are significantly reduced by arsenite (Noreault et al., 2005[280]). Also, arsenite decreases luciferase activity induced by 9-cis-retinoic acid (9cRA), a known RXR ligand, in HepG2 cells loaded with mouse RAR/RXRα heterodimer-dependent retinoic acid response element (RARE)-luciferase reporter as well as ectopic human RXRα (Noreault-Conti et al., 2012[281]).

## 6. Concluding Remarks

The ubiquitous nature of arsenic throughout the environmental ecosystem combined with its powerful toxic properties has rendered it one of the most serious health threats that affects millions of people around the globe.

Arsenic is not confined to its natural mineralogic reservoirs and is inevitably and continuously liberated to the environment both naturally and via several anthropogenic activities. Because the later accounts for much higher rates of release, implementing rigorous regulatory restrictions on such activities is a necessity. 

Initially, arsenic mobilization takes place in the form of water-soluble arsenite and arsenate, and because this is mediated by water, these inorganic species can easily reach different life forms, including humans, where they get biotransformed into more complex organic species. Several arsenic-based compounds and metabolites have been identified with varying toxicity profiles; therefore, arsenic speciation in the potential sources of exposure is required for a meaningful risk assessment.

The fact that the released arsenic cannot be destroyed and just gets transformed from one chemical form to the other may make it more challenging to evade the exposure to its chemical forms which can happen from different sources and through multiple routes. 

Disrupting the metabolic system through interfering with its network of enzymes is one of arsenic multifaceted impacts on the physiological ecosystem throughout the human body. Being a vital component of that system, the impact on the CYPs should have significant consequences especially on xenobiotic activation and/or clearance. The differential toxic behavior of different arsenic compounds entails varying cellular and molecular effects. Studies on different arsenicals have revealed varying species-, tissue-, and/or enzyme- specific effects on the regulation of different CYPs. Further research including interaction between additional arsenic species with more CYP isoforms will absolutely contribute to better understanding of arsenic toxicity, which can then be exploited for developing preventive strategies or serving therapeutic purposes.

## Acknowledgements

This work was supported by Natural Sciences and Engineering Research Council of Canada (NSERC) Discovery Grant [RGPIN 250139] to A.O.S.E. M.A.E. is the recipient of Pharmacy PhD Alumni Graduate Student Scholarship.

## Conflict of interest

The authors declare that they have no conflict of interest.

## Figures and Tables

**Table 1:**
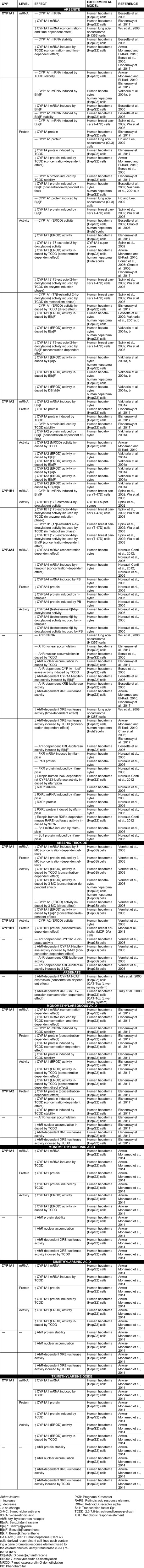
The effect of different arsenic species on the regulation of different cytochrome P450 enzymes (CYPs).
